# Gut microbiota-derived LCA mediates the protective effect of PEDV infection in piglets

**DOI:** 10.1186/s40168-023-01734-4

**Published:** 2024-02-05

**Authors:** Jun-Hong Xing, Tian-Ming Niu, Bo-Shi Zou, Gui-Lian Yang, Chun-Wei Shi, Qing-Song Yan, Ming-Jie Sun, Tong Yu, Shu-Min Zhang, Xi-Ze Feng, Shu-Hui Fan, Hai-Bin Huang, Jun-Hong Wang, Ming-Han Li, Yan-Long Jiang, Jian-Zhong Wang, Xin Cao, Nan Wang, Yan Zeng, Jing-Tao Hu, Di Zhang, Wu-Sheng Sun, Wen-Tao Yang, Chun-Feng Wang

**Affiliations:** College of Veterinary Medicine, Jilin Provincial Engineering Research Center of Animal Probiotics, Jilin Provincial Key Laboratory of Animal Microecology and Healthy Breeding, Engineering Research Center of Microecological Vaccines (Drugs) for Major Animal Diseases, Ministry of Education, Jilin Agricultural University, 2888 Xincheng Street, Changchun, 130118 China

**Keywords:** Infection resistance, Gut microbiota, Macrogenomic, Metabolomic, T cell response

## Abstract

**Background:**

The gut microbiota is a critical factor in the regulation of host health, but the relationship between the differential resistance of hosts to pathogens and the interaction of gut microbes is not yet clear. Herein, we investigated the potential correlation between the gut microbiota of piglets and their disease resistance using single-cell transcriptomics, 16S amplicon sequencing, metagenomics, and untargeted metabolomics.

**Results:**

Porcine epidemic diarrhea virus (PEDV) infection leads to significant changes in the gut microbiota of piglets. Notably, Landrace pigs lose their resistance quickly after being infected with PEDV, but transplanting the fecal microbiota of Min pigs to Landrace pigs alleviated the infection status. Macrogenomic and animal protection models identified *Lactobacillus reuteri* and *Lactobacillus amylovorus* in the gut microbiota as playing an anti-infective role. Moreover, metabolomic screening of the secondary bile acids’ deoxycholic acid (DCA) and lithocholic acid (LCA) correlated significantly with *Lactobacillus reuteri* and *Lactobacillus amylovorus*, but only LCA exerted a protective function in the animal model. In addition, LCA supplementation altered the distribution of intestinal T-cell populations and resulted in significantly enriched CD8^+^ CTLs, and in vivo and in vitro experiments showed that LCA increased SLA-I expression in porcine intestinal epithelial cells via FXR receptors, thereby recruiting CD8^+^ CTLs to exert antiviral effects.

**Conclusions:**

Overall, our findings indicate that the diversity of gut microbiota influences the development of the disease, and manipulating *Lactobacillus reuteri* and *Lactobacillus amylovorus*, as well as LCA, represents a promising strategy to improve PEDV infection in piglets.

Video Abstract

**Supplementary Information:**

The online version contains supplementary material available at 10.1186/s40168-023-01734-4.

## Introduction

Beginning at birth, the host gut is exposed to billions of commensal microbes, which can influence the host’s health and susceptibility to illness [1]. The immune system coevolves to enable the selective implantation of advantageous host bacteria as gut microbes adapt to the gut environment as the body changes. The symbiotic relationship between the gut microbes and their hosts is a delicate balance [2]. The variability of host gut microbes is caused by different diets, environmental factors, genetic factors, etc. [3]. Next-generation sequencing has advanced the study of gut microbial function, and a series of brain/liver/lung/skin-gut-microbiota axis interaction pathways are gradually being elucidated [4–6]. At the same time, intestinal microorganisms play different roles in the development of disease, either facilitating or inhibiting disease development [7, 8]. The main mechanisms by which the intestinal microbiota modulates the immune response, enhances the function of the intestinal barrier, and protects the host from disease are the conversion and recycling of metabolic molecules. Gut-associated diseases such as IBD, intestinal barrier defects, and diarrheal diseases are closely related to intestinal microbiota [9].

PEDV was first isolated in Belgium in 1977 and was classified as a coronavirus [10]. Since then, PEDV has spread around the world, with a mortality rate of up to 90% in piglets, and is characterized by watery diarrhea, dehydration, and vomiting [11]. Animal strains have varying susceptibilities to pathogen infection. The different pathogen resistance levels in model animal C57BL/6 and BALB/c mice are associated with TH1 and TH2 responses [12]. Tibetan pigs, as nonmodel animals, showed a lower susceptibility to PRRSV than large white pigs [13]. Landrace pigs are widely farmed around the world due to their fast growth rate and high lean meat yield. However, compared to Landrace pigs, Min pigs, a premium pig breed from Northeast China, have better disease resistance [14]. The study demonstrated that, under the same rearing conditions within the same pigpen, early transfer of the fecal microbiota from Min pigs to Yorkshire piglets improved the intestinal microbiota structure, intestinal mucosal immunity, and functions associated with intestinal development in Yorkshire piglets [15]. Thus, the unique gut microbes of the Min pig may play an important role in the pathogenic infection process. Although the anti-inflammatory effects of the gut microbes have been demonstrated in adult Min pigs in a DSS-induced colitis model, the composition of the gut microbes and their interaction with viruses during the lactation phase of piglets has not been reported [16].

The biological effects of the gut microbiota involve the conversion and production of metabolites in the host gut. Primary bile acids are synthesized in the liver and then converted by intestinal bacteria via 7α-dehydroxylation to secondary bile acids, including lithobiliary and deoxycholic acids [17]. As research has continued, secondary bile acids have been shown to play multiple roles in controlling the onset of host disease, particularly in preserving the dynamic balance of the intestinal microbiota and the mucosal immune system [18]. The farnesol X receptor (FXR, NR1H4) and GPBAR1 (also known as TGR5) function as important bile acid receptors and are involved in signal transduction pathways [19]. This study elucidates that the gut microbiota converts primary bile acids into secondary bile acids and alleviates autoimmune uveitis by modulating dendritic cells through the TGR5 receptor [20]. At the same time, bile acid metabolites can directly regulate Th17 and Treg balance and RORγ+ regulatory T-cell homeostasis to control the host immune response [21, 22]. Thus, gut microbial-mediated metabolites exerting specific regulatory functions are also important in gut microbial-host interactions.

We hypothesized that the composition of the gut microbiota could be one of the factors contributing to the differential disease resistance observed in different pig breeds. Therefore, in this study, we utilized fecal microbiota transplantation (FMT) and a multi-omics approach combining single-cell RNA sequencing (scRNA-seq), 16S amplicon sequencing, metagenomic sequencing, and untargeted metabolomics to investigate the crosstalk between the gut microbiota, metabolites, and intestinal immunity. Our aim was to unravel the mechanisms underlying the modulation of host health by the gut microbiota.

## Methods

### Cells, bacteria, and virus

Vero cells and IPEC-J2 cells were cultured in DMEM (SH30809.01, HyClone, USA) supplemented with 10% fetal bovine serum (10099141C, Gibco, USA) at 37 °C and 5% CO_2_.

*Lactobacillus reuteri* and *Lactobacillus amylovorus* were purchased from the China Center of Industrial Culture Collection (CICC), and the numbers of the strains were CICC6123 and CICC6090, respectively. These two strains were cultured in MRS medium for 20 h under anaerobic conditions at 37 °C.

PEDV CV777 was kept in this laboratory and prepared using Vero cells. The viral units used in this study were 10^3.5^ PFU ml^−1^.

### Animals and experimental design

Min pigs and Landrace pigs were obtained from Jilin Academy of Agricultural Sciences, Jilin Province. Three days after the intake of colostrum, piglets were weaned, followed by artificial feeding of milk. All piglets tested negative for PEDV. The diet and environmental conditions were kept consistent for all piglets, and each experimental group was isolated in a separate room. To ensure consistent and standardized infection, all inoculation experiments were performed by orally gavaging 1 ml of PEDV (10^3.5^ PFU ml^−1^) to piglets at 11 days of age. The day of inoculation was considered as day 0, and the piglets’ weight changes, survival rate, and occurrence of diarrhea were observed over the following 14 days. The timeline of the animal experiments (A–D) are presented in Fig. S1. Animal experiments are divided into the following four parts.

#### Comparative experiment between Min pigs and Landrace pigs regarding PEDV infection

Min pigs and Landrace pigs (11 days old) were randomly divided into four groups (*n*=10): M-CON group, Min pigs challenged with PBS; M-PEDV group, Min pigs challenged with PEDV; L-CON group, Landrace pigs challenged with PBS; L-PEDV group, Landrace pigs challenged with PEDV.

#### FMT experiment

Min pigs and Landrace pigs (3 days old) were randomly divided into three groups. From −8 to −3 days, the groups received combination antibiotic treatment. From −3 days to day 0, FMT was conducted. On day 0, the piglets were infected with PEDV. NC group (*n*=5), Landrace pigs without FMT treatment; L donor group (*n*=8), Landrace pigs treated with FMT from Landrace pigs; M donor group (*n*=8), Landrace pigs treated with FMT from Min pigs.

#### Probiotic validation experiment

Min pigs and Landrace pigs (3 days old) were randomly divided into five groups (*n*=5). From −8 to−3 days, the groups received combination antibiotic treatment. From −3 days to day 0, supplementation of *Lactobacillus amylovorus* (10^10^ CFU) and *Lactobacillus reuteri* (10^10^ CFU) was administered. On day 0, the piglets were infected with PEDV. To evaluate the colonization efficacy of probiotics, pigs were administered *Lactobacillus reuteri* and *Lactobacillus amylovorus* subsequent to antibiotic treatment. Quantitative assessments of microbial abundance were carried out on days 3, 7, and 14 following administration. Am group, pigs gavaged with *Lactobacillus amylovorus*; Re group, pigs gavaged with *Lactobacillus reuteri*; Am/Re group, pigs gavaged with *Lactobacillus amylovorus* and *Lactobacillus reuteri*; PBS group, Min pigs gavaged with PBS; NC group, normal control group.

#### Metabolite validation experiment

Min pigs and Landrace pigs (3 days old) were randomly divided into five groups (*n*=5). From −8 to −3 days, the groups received combination antibiotic treatment. From −3 days to day 0, supplementation of metabolites was conducted. Specifically, 0.02% of DCA (434139, Sigma, USA) and LCA (83443, Sigma, USA) were added to the milk formula for feeding. On day 0, the piglets were infected with PEDV. DCA group, pigs gavaged with DCA; LCA group, pigs gavaged with LCA; L/DCA group, pigs gavaged with DCA and LCA; PBS group, Min pigs gavaged with PBS; NC group, normal control group.

Stool consistency scores range from 1 to 4 (0 = normal, 1 = soft, 2 = thick, and 3 = completely liquid). Fecal samples were collected on day 8 post-challenge and were immediately stored at −80 °C for total DNA extraction and sequencing and metabolite analysis. On day 8 following the challenge, piglet jejunal tissues were obtained, processed, and examined for single-cell sequencing for the LCA treatment experiments, and replicate trials were set up for all experimental groups to ensure adequate sample sizes.

### Antibiotic treatment and FMT

Ampicillin (1 mg/ml) (A8180, Solarbio, China), streptomycin (1 mg/ml) (S8290, Solarbio, China), vancomycin (0.25 mg/ml) (V8050, Solarbio, China), and neomycin (1 mg/ml) (N8090, Solarbio, China) were added to the milk and fed for 5 days to eliminate as much as possible the gut microbial background of the piglets, followed by FMT or feeding of bacteria or metabolites. Feces were collected on day 8 from the uninfected group of Min pigs and Landrace pigs and treated as described previously. Briefly, fecal samples were placed in a sterile N2 incubator and mixed thoroughly. Then, cell filters were inserted to collect the bacteria and centrifuged at 600×g for 10 min. The samples were then added to 10% glycerol and stored at −80 °C [23]. Prior to FMT, PBS was used instead of glycerol, and the number of viable bacteria was subsequently counted by methylene blue staining. Two milliliters of the bacterial solution were mixed into the milk and fed to the piglets for three consecutive days, and the PEDV challenge occurred on day 0 by gavage.

### 16S rDNA sequencing and data analysis

Genomic DNA was extracted from fecal samples using cetyltrimethylammonium bromide (CTAB). DNA extraction quality was evaluated on a 1% agarose gel, and DNA quantification was performed using a UV spectrophotometer. The DNA was diluted to a concentration of 1 ng/μl using sterile water as a standard for subsequent PCR amplification. To ensure the accuracy and efficiency of amplification, PCR was carried out on the V3–V4 region using certain primers with barcodes based on the chosen sequencing region utilizing the diluted genomic DNA as a template. The primer sequences were as follows: forward primer 5′-GTACTCCTACGGGAGGCAGCA-3′; reverse primer 5′-GTGGACTACHVGGGTWTCTAAT-3′. The PCR products were detected by electrophoresis using a 2% concentration agarose gel, and the PCR products that passed the test were purified by magnetic beads and quantified by enzyme labeling. Following complete mixing and homogenization of the samples in accordance with the concentration of the PCR product, the target band products were recovered after the PCR products were identified using 2% agarose gel electrophoresis. Libraries were constructed using the TruSeq® DNA PCR-Free Sample Preparation Kit (Illumina, USA). The libraries were quantified by Qubit (Thermo Scientific) and Q-PCR and then sequenced using NovaSeq6000 (KapaBiosciences, Woburn, MA, USA).

The samples were sequenced on an Illumina NovaSeq platform in accordance with the manufacturer’s instructions. Paired-end readings were assigned to samples based on their unique barcode and were then shortened by removing the barcode and primer sequence. Paired-end reads were merged using FLASH (V1.2.7, http://ccb.jhu.edu/software/FLASH/). Quality filtering of the raw reads was performed under specific filtering conditions to obtain high-quality clean tags according to fqtrim (v0.94) (CNIC, China). Chimeric sequences were filtered using Vsearch software (v2.3.4) (BILS, Sweden). After dereplication using DADA2, the beta diversity and alpha diversity were computed by normalizing to identical sequences generated at random. The feature abundance was then normalized using the relative abundance of each sample in accordance with the SILVA (Release 138, https://www.arb-silva.de/documentation/release138/) classifier. Alpha diversity is used to analyze the complexity of species diversity for a sample using 5 indices, all of which were calculated with QIIME (V1.9.1, http://qiime.org/scripts/split_libraries_fastq.html) in our data. The beta diversity was estimated using QIIME, and graphics were created using the R package (v3.5.2). For each representative sequence, the feature sequences were annotated with the SILVA database using Blast. Venn diagrams, principal coordinate analysis (PCoA), phylogenetic analysis, correlation heatmaps, and correlation network plots were generated using the R package (v3.5.2).

### Metagenome assembly and functional annotations

Clean data were assembled and analyzed using MEGAHIT software (v1.0.4), and the assembled scaffolds were then broken from the N linkage to give N-free scaftigs [24, 25]. Unigenes were matched against functional databases using DIAMOND software (vO.9.9.110, https://github.com/bbuchfink/diamond/) [26, 27]. Functional databases included the KEGG database (version 2018-01-01, http://www.kegg.jp/kegg/) [28], eggNOG database (version 4.5, http://eggnogdb.embl.de/#/app/home) [29], and CAZy database (version 201801, http://www.cazy.org/) [30]. The best BLAST hit results were selected for subsequent analysis [31]. The relative abundance of the various functional levels was calculated based on the comparison results [32]. Tables representing the number of genes in each sample at each taxonomic level were generated using the functional annotation results and gene abundance tables. The number of genes in a given sample for a certain function is equal to the number of genes whose abundance is not 0 among those annotated for that function. Differential function was assessed from abundance tables at each taxonomic level.

### LC‒MS/MS

To identify the metabolites in piglet feces, 100 mg of fecal sample was placed in an EP tube and vortexed with 500 μl of 80% aqueous methanol. After 5 min in an ice bath, the samples were centrifuged at 15,000 × g for 20 min at 4 °C. The supernatant was diluted with mass spectrometry-grade water to 53% methanol. The supernatant was collected by centrifugation at 15,000 × g for 20 min at 4 °C and analyzed by LC‒MS. In addition, BA (100uM) was added during the culture of *Lactobacillus reuteri* and *Lactobacillus amylovorus*. After 12 h of culture, the supernatant was separated for LC‒MS analysis [33].

The selected m/z scan range was 100–1500. The ESI source was set up as follows: spray voltage, 3.5 kV; sheath gas flow rate, 35 psi; aux gas flow rate, 10 l/min; capillary temp, 320 °C; S-lens RF level, 60; aux gas heater temp, 350 °C; and polarity, positive, negative. MS/MS secondary scanning was used for data-dependent scanning. The downstream data (raw) files were imported into CD 3.1 library search software for processing and simple screening of each metabolite for parameters such as retention time and mass-to-charge ratio, and then a retention time deviation of 0.2 min and mass deviation of 5 ppm were set for peak alignment of different samples for more accurate identification. Subsequently, information such as mass deviation of 5 ppm, signal intensity deviation of 30%, signal-to-noise ratio of 3, minimum signal intensity, and summed ions was set for peak extraction. The peak areas were quantified, integrated for the target ions, predicted by molecular ion peaks and fragment ions for molecular formulae, and compared with the mzCloud (https://www.mzcloud.org/), mzVault, and Masslist databases. Background ions were removed using blank samples, and the raw quantification results were normalized to give the final identification and relative quantification of metabolites. The data processing is partly based on the Linux operating system (CentOS version 6.6) and R and Python software.

The identified metabolites were annotated using the KEGG database (https://www.genome.jp/kegg/pathway.html), the HMDB database (https://hmdb.ca/metabolites), and the LIPIDMaps database (http://www.lipidmaps.org/). To obtain VIP values for each metabolite, the data were converted using the metabolomics data processing program metaX [34] and subjected to principal component analysis (PCA) and partial least square discriminant analysis (PLS-DA). The univariate analysis section was based on a *t* test to calculate the statistical significance (*P* value) of each metabolite between the two groups and to calculate the fold change (FC-value) of the difference between the metabolites in the two groups. The default criteria for differential metabolite screening were VIP >1, *P* value <0.05, and FC ≥2 or FC ≤0.5. Volcano plots were drawn with the R package ggplot2, which can combine the three parameters of VIP value, log2 (FoldChange), and -log10 (*P* value) of metabolites to screen for metabolites of interest. Correlation analysis (Pearson’s correlation coefficient) between different metabolites was performed using R. Statistical significance was achieved using R. A *P* value < 0.05 was considered statistically significant, and correlation plots were plotted using the corrplot software package in R. Bubble plots were plotted using the R package ggplot2, and the KEGG database was used to examine metabolite function and metabolic pathways, which were considered enriched when *x*/*n*>*y*/*n* and significantly enriched when the *P* value for the metabolic pathway was <0.05.

### Single-cell RNA sequencing

The piglet jejunum tissues were cut into 0.5 mm^2^ pieces and isolated into single cells in a mixture of 0.35% collagenase IV (C4-22, Sigma, USA) and 120 units/ml DNase I (D7076, Beyotime, China). Cells were filtered through a 40-μm cell filter and isolated using 40% Percoll (10317837, Cytiva, USA) and 80% Percoll in a centrifuge at 20 °C and 500 × g for 20 min. The middle layer was then carefully aspirated into a 15-ml centrifuge tube and washed once with PBS at 4 °C and 400 × g for 5 min. The supernatant was discarded, and 1 ml of RPMI 1640 medium containing 3% FBS was used for resuspension to prepare a single cell suspension and counted at 20-fold dilution. Cells were stained using PE-labeled anti-pig CD3 (561485, BD, USA) and subsequently collected by positive screening in LS Columns (130-122-729, Miltenyi Biotec, Germany) using Anti-Phycoerythrin (PE) MicroBeads (130-048-801, Miltenyi Biotec, Germany). Single-nuclei suspensions were loaded onto 10x chromium to capture 8000 single cells according to the manufacturer’s instructions for the 10X Genomics Chromium Single-Cell 3’ kit (V3). The following cDNA amplification and library construction steps were performed according to the standard protocol. Libraries were sequenced on an Illumina NovaSeq 6000 sequencing system (paired-end multiplexing run, 150 bp) by LC-Bio Technology Co., Ltd. (Hangzhou, China) at a minimum depth of 20,000 reads per cell.

Sequencing results were demultiplexed and converted to FASTQ format using Illumina bcl2fastq software (version 5.0.1). Sample demultiplexing, barcode processing, and single-cell 3’ gene counting were performed by using the Cell Ranger pipeline (https://support.10xgenomics.com/single-cell-geneexpression/software/pipelines/latest/what-iscell- ranger, version 6.1.1), and scRNA-seq data was aligned to the Sscrofa Ensemble 11.1 reference genome. The Cell Ranger output was loaded into Seurat (version 3.1.1) for dimensional reduction, clustering, and analysis of scRNA-seq data. Overall, 25,000 cells passed the following quality control thresholds: all genes expressed in less than three cells (default parameters, 1 cell) were removed, as well as the number of genes expressed per cell > 500 as the lower cut-off and <5000 as the upper cut-off, UMI counts less than 500, and the percent of mitochondrial-DNA derived gene-expression <25%. To visualize the data, we further reduced the dimensionality of all cells using Seurat and used t-SNE to project the cells into 2D space

### Histopathology and IFA assessment

Piglet jejunum tissues were fixed in 4% paraformaldehyde and subsequently paraffin-embedded on the 8th day after infection with PEDV. The tissue was dehydrated through a gradient series of alcohol and then sectioned. At least 2 tissue sections (3 μm) of each sample were stained with hematoxylin-eosin (HE) and finally analyzed in the laboratory using an inverted fluorescence microscope (Leica Microsystems, Germany). The other section was processed and stained with anti-PEDV-S antibody (1:1000) (24B9, LanDuBio, China) and subsequently incubated with FITC-labeled goat anti-mouse antibody (1:1000) (GB22301, Servicebio, China). Subsequently, the cells were stained with DAPI for 5 min, covered with an anti-fluorescent attenuator (P0126, Beyotime, China) and observed under a microscope in the dark (Leica Microsystems, Germany). The images are scanned at magnifications of 40× and 200×.

Scoring for the small intestinal changes was based on the grade of epithelial cell desquamation [35, 36], which was measured as follows: 0 = normal (no desquamation), 1 = mild (a few desquamated cells of tip villous epithelium), 2 = moderate (desquamation of upper villous epithelium), 3 = marked (desquamation of lower villous epithelium), and 4 = severe (desquamation of crypt epithelium). Nine regions were randomly selected on the intestinal tissue sections to determine the average lesion change. The mean fluorescence intensity was estimated by ImageJ (1.8.0.345) in nine randomly selected regions of the intestinal fluorescence image.

### Flow cytometry

Jejunum tissues and mesenteric lymph nodes (MLN) of piglets were collected on the 8th day after infection with PEDV. After processing and digesting the piglet jejunum into a single cell suspension using collagenase IV, cells were isolated using the Percoll method (40% and 80% Percoll) (10317837, Cytiva, USA) and adjusted to 10^6^ cells per sample. MLN were processed after grinding to separate cells using a 40-μm cell strainer and adjusted to 10^6^ cells. To avoid nonspecific binding, 10^6^ cells were closed for 40 min under light-proof conditions using 1 μg of Fixable Stain 780 (BD, USA). Subsequent staining was carried out using the cell reactive dye Zombie NIR™ (423105, BioLegend, USA) for 20 min in the dark. After washing with FACS buffer solution (PBS containing 0.1% BSA and 0.08% sodium azide) 2 times, the samples were stained with PE-labeled anti-pig CD3 (561485, BD, USA) and FITC/PE-labeled anti-pig CD8 (551303/559584, BD, USA). After incubation at 4 °C for 30 min, FACS buffer was used for washing twice, and the cells were centrifuged at 500×g for 5 min. Next, the cells were treated with fixation and permeabilization solution (51-2090KZ, BD, USA) and stained intracellularly with APC-Cy7-labeled Granzyme A (507220, Biolegend, USA), FITC/PerCP/Cyanine5.5-labeled Granzyme B (372212, Biolegend, USA) and PerCP/Cyanine5.5-labeled Perforin (353313, Biolegend, USA) for intracellular staining. After incubation at 4 °C for 30 min, FACS buffer was used for washing twice, followed by centrifuging at 500×g for 5 min. Finally, 200 μl of PBS was mixed evenly, and samples were subjected to flow cytometry. Macrophages were labeled with PE-Cy7-labeled anti-pig CD3 (561477, BD, USA) and PE-labeled anti-pig CD163 (XK3763066A, Invitrogen, USA), and dendritic cells were labeled with PE-Cy7-labeled anti-pig CD3 (561477, BD, USA), anti-pig SLA-II (MA5-28503, Invitrogen, USA), and the secondary antibody goat anti-mouse pAb (PE-Cy®5.5 Conjugate) (2142776, Invitrogen, USA) in the same way as for T-cell surface staining. The data was analyzed by FlowJo 10.8.1 software.

### Enzyme‑linked immunosorbent assay

PEDV (3378701), PEDV-specific IgA (3634302), pig IL-2 (042101), and SLA-I (044101) were detected using an enzyme-linked immunosorbent assay (ELISA) kit (MEIMIAN, China) according to the manufacturer’s instructions.

### IPEC-J2 cell treatments

When IPEC-J2 cells reached approximately 70% confluence, the medium was changed to 10/20 μM LCA (83443, Sigma, USA) solution, 2 μM GW4064 (HY-50108, MedChemExpress, USA), 5 μM CCDC (SD2384, Beyotime, China), 2 μM SBI-115 (HY-111534, MedChemExpress, USA), or 2 μM Gly-β-MCA (HY-114392, MedChemExpress, USA) (the culture medium for the blank group was replaced with basal DMEM) and incubated for 12 h. Each sample was adjusted to 10^5^ cells after incubation, and a portion was treated with SLA-I (XK3759109, Invitrogen, USA) antibody for 20 min while shielded from light. This fraction was then analyzed using a flow cytometry device. The expression of SLA-I in the remaining cells was then determined by ELISA after the other cells were ultrasonically lysed.

### CTL cytotoxicity assay

On day 8 after piglets were challenged with PEDV, jejunal monocytes were stained using PE-labeled anti-pig CD8 (559584, BD, USA), and cells were subsequently collected as effector cells by positive screening in LS Columns (130-122-729, Miltenyi Biotec, Germany) using Anti-Phycoerythrin (PE) MicroBeads (130-048-801, Miltenyi Biotec, Germany). IPEC-J2 cells were grown to 70% confluence, the medium was discarded, and 10 μM LCA, 2 μM SBI-115 (HY-111534, MedChemExpress, USA), or 2 μM Gly-β-MCA (HY-114392, MedChemExpress, USA) was added. IPEC-J2 cells were subsequently infected with PEDV (10_3.5_ PFU ml^−1^) for 8 h. After coculture of the effector and target cells for 4 h, the supernatant was collected, and the cytotoxicity of each group was assayed using the LDH Cytotoxicity Assay Kit (C0017, Beyotime, China) according to the manufacturer’s instructions.

### T-cell proliferation and IL-2 assay

Jejunum tissues of piglets were collected on the 8th day after infection with PEDV. After the pigs’ jejunums had been digested, CD8 (559584, BD, USA) and CD4 (PE-conjugated, 559586, BD, USA) T cells were isolated using magnetic beads, and the cell concentration was increased to 5×10^5^/ml using RPMI 1640 containing 10% FBS. Cells were stained with carboxyfluorescein succinimidyl ester (CFSE) (4238011, Biolegend, USA), incubated for 10 min at 37 °C, washed and transferred to cell culture plates, activated by adding 5 μg/ml anti-CD3 (BE0001-2, BioX cell, USA) and 2 μg/ml anti-CD28 (BE0248, BioX cell, USA) mAbs, and treated with LCA (10/20 μM). Cells were incubated in a cell incubator at 37 °C, and cell proliferation was measured at 12-h intervals. Granzyme A, granzyme B, and perforin expression was also measured at 24 h. In addition, cell supernatants were collected at 24 h to detect IL-2 expression using ELISA.

### Statistical analysis

The animals were randomly assigned to the experimental and control groups. All data are expressed as the mean ± SD. Statistical analysis was performed using one-way ANOVA and *t* tests with GraphPad Prism 8.0 (GraphPad software). The *P* values are indicated as follows: *P < 0.05; **P < 0.01; ***P < 0.001; ****P < 0.0001.

## Results

### Differences in PEDV infection in Min pigs and Landrace pigs

**Fig. 1:**
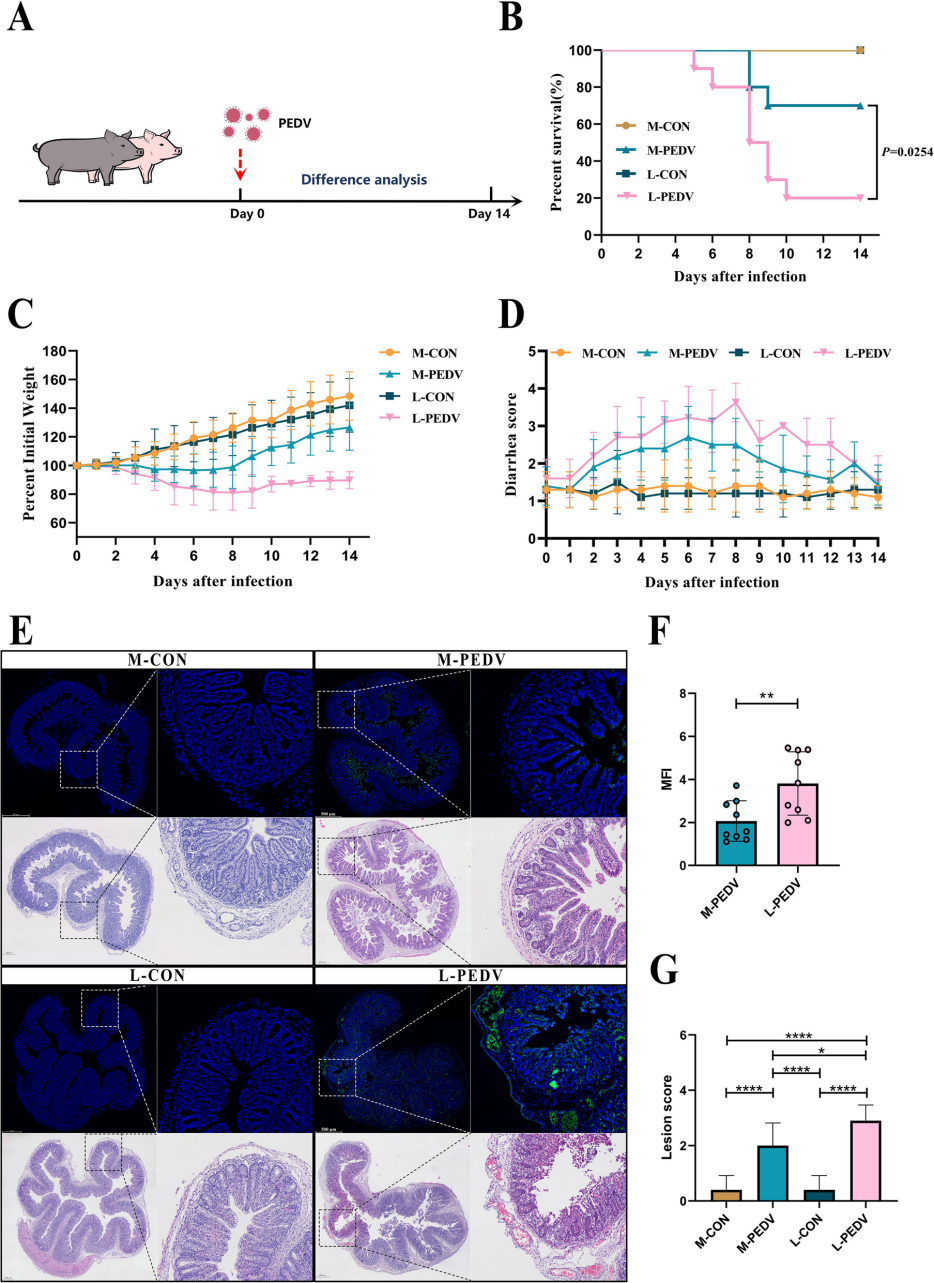
Differences in PEDV infection between Min pigs and Landrace pigs. **A** PEDV infection model in Min pigs and Landrace pigs (*n*=10/group) (M-CON, Min pigs challenged with PBS; M-PEDV, Min pigs challenged with PEDV; L-CON, Landrace pigs challenged with PBS; L-PEDV, Landrace pigs challenged with PEDV). Comparative analysis of survival (**B**), weight loss (**C**), and diarrhea (**D**) in each group of piglets. **E** Comparison of histopathological changes (H&E staining) and PEDV viral load in the jejunum of piglets on day 8 post-infection. The left image is scanned at a magnification of 40×, and the right image is scanned at a magnification of 200×. **F** Analysis of average fluorescence intensity. **G** Histopathological scoring of tissues. The results are presented as the means ± SD, and statistical significance was calculated by *t* tests for two groups and one-way ANOVA for four groups. *P < 0.05; **P < 0.01; ****P < 0.0001

We created a model of PEDV infection in breastfeeding piglets to ascertain whether there are variations in responses to virus infection between Min pigs and Landrace pigs (Fig. 1A). The piglets were challenged by gavage after 3 days of acclimatization, and the difference in infection between Min pigs and Landrace pigs was evaluated over the next 14 days. We found that compared to the L-PEDV pigs, the M-PEDV pigs showed greater resistance to weight change (Fig. 1C) and survival (Fig. 1B), especially at day 8, and had less diarrhea (Fig. 1D) than the L-PEDV pigs. Because PEDV targets the intestine and replicates in intestinal cells, we examined PEDV damage to the intestine and the viral load in piglets of both strains (Fig. 1E) and assessed epithelial cell desquamation (Fig. 1G) and the mean fluorescence intensity (Fig. 1F). The results showed that the intestinal epithelial cell necrosis of the L-PEDV pigs was severe, and the intestinal epithelium was entirely peeled off. In contrast, the M-PEDV pigs showed the loss of modest intestinal epithelial cells, and the intestinal villi was intact. Additionally, the gut of M-PEDV pigs displayed a lower virus load, in contrast to the L-PEDV pigs, whose intestines were rich in excess PEDV.

### There are differences in the Min pig and Landrace pig gut microbes

**Fig. 2:**
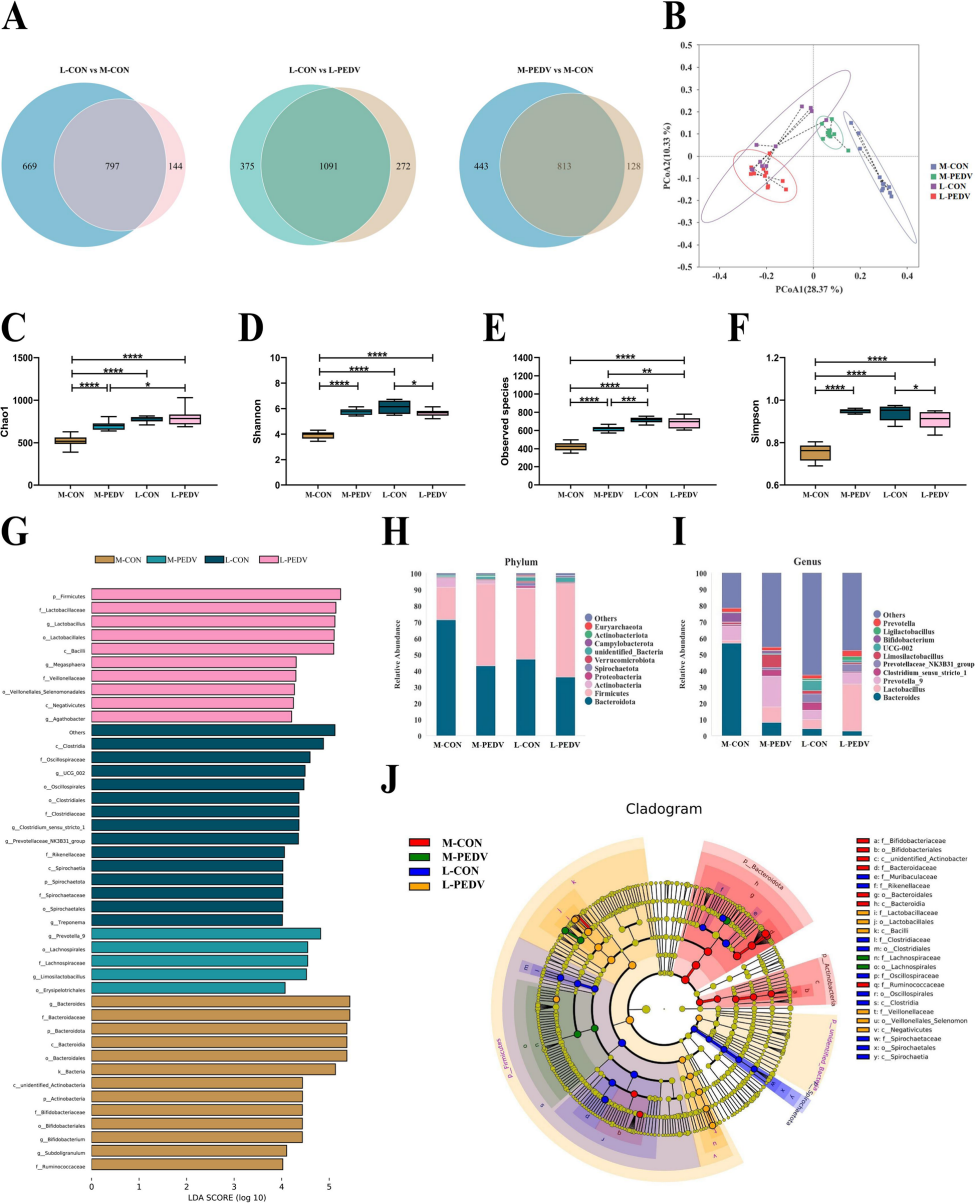
Differences in the gut microbes of Min pigs and Landrace pigs. **A** Comparative analysis of intestinal microbial OTUs of M-CON, M-PEDV, L-CON, and L-PEDV pigs. **B** PCoA analysis based on weighted UniFrac distances to identify microbial structural changes. Comparative analysis of Chao1 (**C**), Shannon (**D**), observed species (**E**), and Simpson (**F**) indices among different groups. The top 10 species at the phylum (**H**) and genus (**I**) levels among different groups. **G** LEfSe counted species with LDA scores greater than 4 between groups and screened for statistically significant differences in biomarkers. **J** Analysis of LEfSe evolutionary branches using differential biomarkers. The results are presented as the means ± SD, and statistical significance was calculated by one-way ANOVA. *P < 0.05; **P < 0.01; ***P <0.001; ****P < 0.0001

We assume that the Min pigs and Landrace pigs are colonized by different microbes during breastfeeding, and PEDV infections may further affect the gut microbes. We used 16S rDNA analysis to compare the gut microbes of Min pigs and Landrace pigs to confirm this notion. The 1256 OTUs of the M-CON pigs in the infected group were significantly fewer than the 941 OTUs of the Landrace pigs. Following infection, the number of OTUs for M-PEDV pigs grew, while the number of OTUs for L-PEDV pigs decreased (Fig. 2A). To assess alpha diversity, the Chao1 (Fig. 2C), Shannon (Fig. 2D), observed species (Fig. 2E), and Simpson (Fig. 2F) indices were applied. We discovered that the diversity of intestinal bacteria in M-CON pigs was significantly lower than that in L-CON pigs but that it significantly increased following PEDV infection. In addition, the diversity of gut microbes of L-PEDV pigs is not affected by PEDV infection. At the same time, PCoA was used for PCoA based on weighted UniFrac distances. The composition of the M-CON pig gut microbes was different from that of the Landrace pigs, and there were more obvious community differences after PEDV infection (Fig. 2B). In addition, the relative abundances of the species of each group were analyzed at the phylum level and genus level.

At the phylum level, we found that more *Bacteroidota* and *Actinobacteria* were enriched in M-CON pigs than in L-CON pigs. The abundance of *Firmicutes* was significantly increased in M-PEDV pigs (Fig. 2H). At the genus level, the M-CON pigs had more *Bacteroides* than the L-Con pigs, and the abundance of *Bacteroides* in the M-PEDV pigs was greatly decreased. In addition, the M-PEDV and L-PEDV pigs had a marked increase in *lactobacillus* abundance, which may interact with PEDV infection (Fig. 2I). To identify the relationship between bacteria, we analyzed the interaction of the genus (top 30) (Fig. S2A) and system development and evolution (top 100) (Fig. S2B). We used LEfSe to examine the distribution of various species (Fig. 2G) and system development (Fig. 2J) to determine the distinct biomarkers of each group. M-CON pigs contained *Bacteroides*, *Bifidobacterium*, *Subdoligranulum*, and *Lacticaseibacillus*. *Prevotella-9* and *Limosilactobacillus* were enriched in the M-PEDV pigs. At the same time, the L-PEDV pigs were enriched in the beneficial bacteria *Lactobacillus* and *Agathobacter*, as well as *Megasphara*.

### FMT of the gut microbes of Min pigs can protect piglets against PEDV

**Fig. 3:**
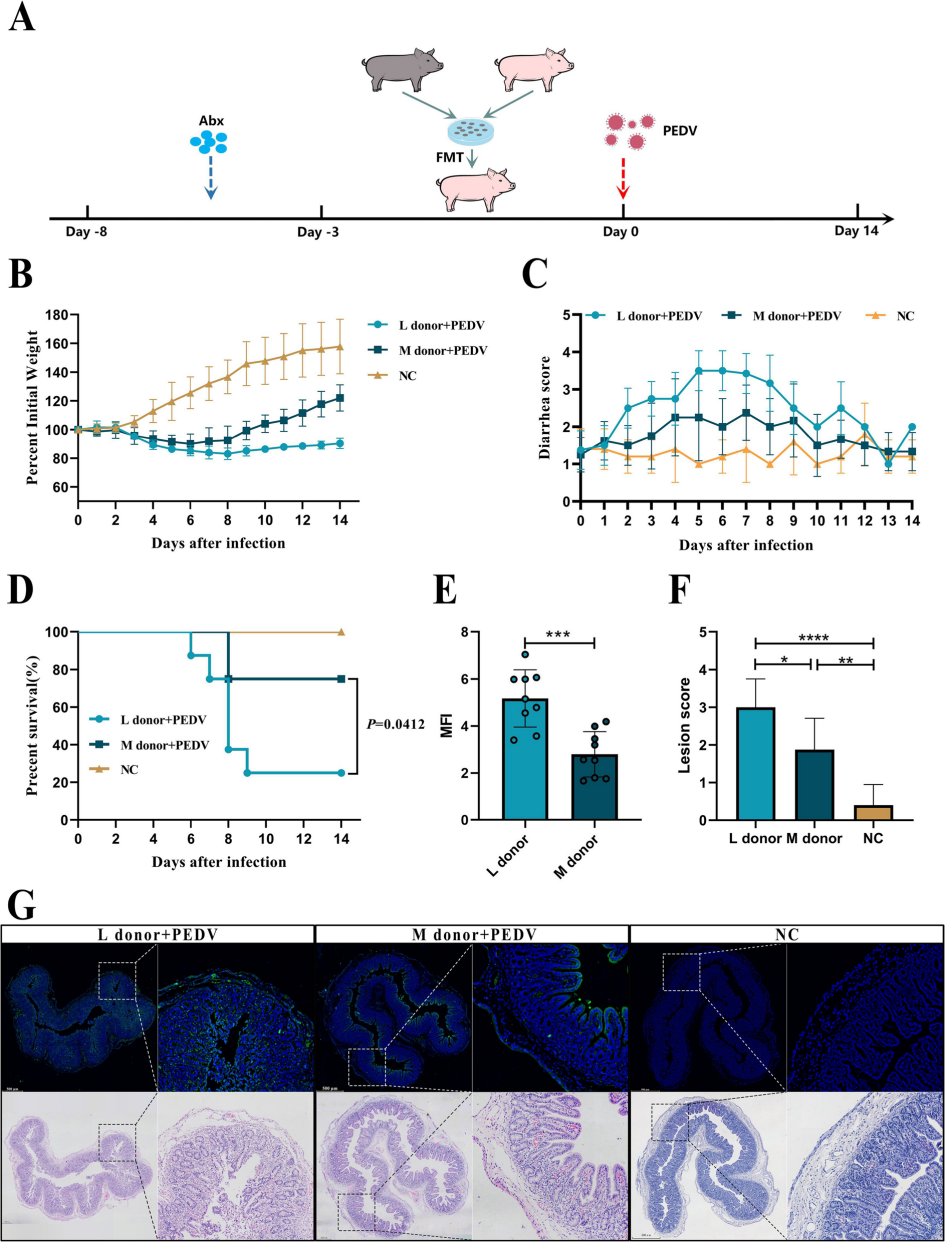
Gut microbes mediate the protective effect of PEDV in Min pigs. **A** FMT model in Min pigs and Landrace pigs (NC, Landrace pigs without FMT treatment, *n*=5; L donor, Landrace pigs treated with FMT from Landrace pigs, *n*=8; M donor, Landrace pigs treated with FMT from Min pigs, *n*=8). Comparative analysis of survival (**D**), weight loss (**B**), and diarrhea (**C**) in each group of piglets. (**G**) Comparison of histopathological changes (H&E staining) and PEDV viral load in the jejunum of piglets on day 8 post-infection. The left image is scanned at a magnification of 40×, and the right image is scanned at a magnification of 200×. **E** Analysis of average fluorescence intensity. **F** Histopathological scoring of tissues. The results are presented as the means ± SD, and statistical significance was calculated by *t* tests for two groups and one-way ANOVA for four groups. *P < 0.05; **P < 0.01; ***P < 0.001; ****P < 0.0001

The FMT model was established next. We utilized a combination of antibiotics to exhaust the bacteria and 16S rDNA sequencing to confirm the exhaustion effectiveness to remove the backdrop of mammary piglet intestinal microbes. Following antibiotic treatment, the variety of species (Fig. S3B, C) and the number of OTUs (Fig. S3A) among pig intestinal bacteria drastically decreased. The microbiota structure changed concurrently (Fig. S3D), and the diversity and abundance dropped (Fig. S3E, F), demonstrating that antibiotic treatment eliminated the potential effects. Then, we transplanted the gut microbes of the Min pigs and Landrace pigs into Landrace pigs and then challenged them with PEDV (Fig. 3A). We discovered that the M donor pig displayed improved anti-infectivity when compared to L donor pigs, which was represented by a slight weight loss (Fig. 3B) and diarrhea (Fig. 3C), as well as higher survival rates (Fig. 3D). At the same time, the intestinal injury (Fig. 3G, F) of the M donor pigs and the PEDV virus load (Fig. 3G, E) were lower than those of the L donor pigs. The PEDV fluorescence strength and epithelial cell desquamation also proved the protection provided by Min pig gut microbes.

Min pigs are rich in *Lactobacillus reuteri* and *Lactobacillus amylovorus* after infection with PEDV

**Fig. 4:**
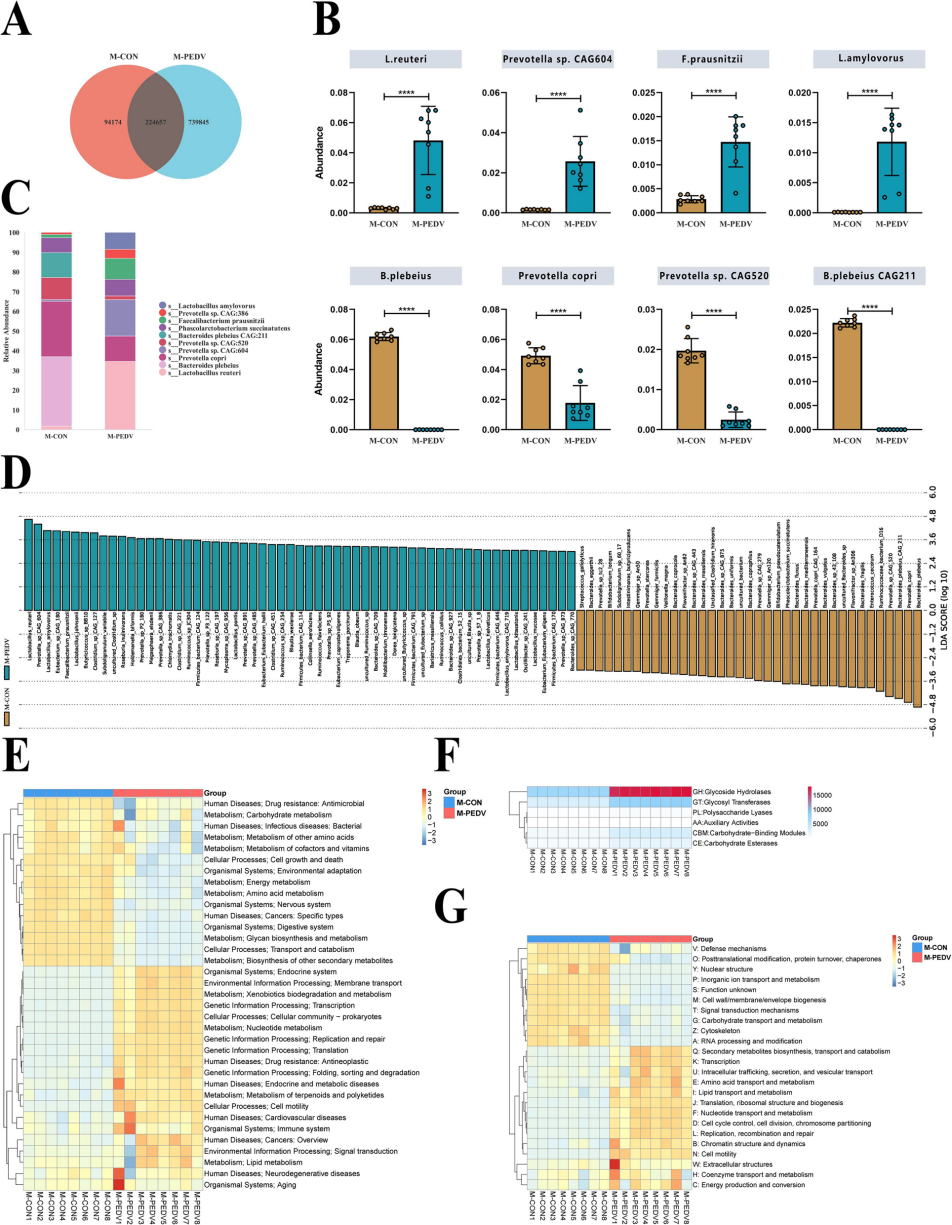
Metagenomic analysis of gut microbiota in M-CON and M-PEDV piglets. **A** Gene number distribution of M-CON pig and M-PEDV pig gut microbes (*n*=8/group). **B** Analysis of significantly upregulated (top 4) and downregulated (top 4) species in the abundance table. **C** Species comparison analysis (top 10). **D** LEfSe analysis (LDA score > 4). Functional annotation and abundance information of the samples in the KEGG (**E**) and eggNOG (**G**) databases and clustering at the level of functional differences. **F** Functional annotation and statistics of samples in the CAZy database. The results are presented as the means ± SD, and statistical significance was calculated by one-way ANOVA. ****P < 0.0001

We analyzed the different species among the virus-infected and control groups in the same period through metagenomics. After the genes were assembled and screened, we found that the Min pigs infected with PEDV had a significantly increased number of gut microbe genes (Fig. 4A). We then calculated the relative abundances of the bacteria after species annotation. The abundances of *Lactobacillus reuteri*, *Lactobacillus amylovorus*, *Prevotella* sp. *CAG:604*, *Phasolarctobacterium succinatutens*, and *Faecalibacterium prausnitzii* all increased following virus infection, whereas the abundances of *Bacteroides plebeius*, *Prevotella copri*, *Prevotella* sp. *CAG:520*, *Bacteroides plebeius CAG:211*, and *Phascolarctobacterium succinatutens* were depleted (Fig. 4C). In addition, cluster statistics (Fig. S4A), Metastat (Fig. 4B), and LEfSe (Fig. 4D) were used to analyze the differences in species. The comparison calculation results are the same, and the superior bacteria represented by *Lactobacillus reuteri* and *Lactobacillus amylovorus* are significantly enriched during virus infection. Additionally, functional annotation analysis of the KEGG database revealed that following PEDV infection, there was an increased enrichment of genes in metabolic pathways (Fig. S4B). Moreover, differential functional analysis identified nucleotide metabolism, metabolism of terpenoids and polyketides, and lipid metabolism, as the predominant enriched pathways within the metabolic pathways (Fig. 4E). Nucleotide metabolism and lipid metabolism were likewise the primary enrichment pathways after comparison utilizing the -EggNOG database (Fig. 4G). Furthermore, a comparison with the CAZy database revealed increased function of glycoside hydrolases and GLYcosyl transferases (Fig. 4F).

*Lactobacillus reuteri* and *Lactobacillus amylovorus* provide protection against PEDV

**Fig. 5:**
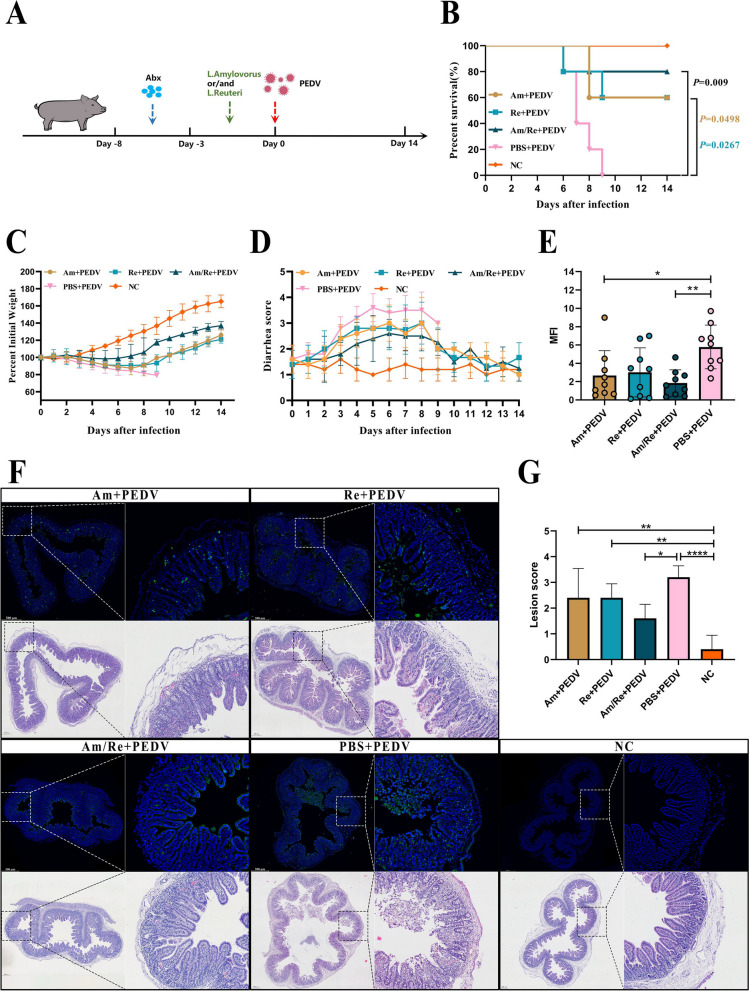
Efects of *Lactobacillus reuteri* and *Lactobacillus amylovorus* on PEDV infection in piglets. *Lactobacillus reuteri* and *Lactobacillus amylovorus* alleviate PEDV infection. **A** Protective model of *Lactobacillus reuteri* and *Lactobacillus amylovorus* in piglets (Am, Min pigs gavaged with *Lactobacillus amylovorus*; Re, Min pigs gavaged with *Lactobacillus reuteri*; Am/Re, Min pigs gavaged with *Lactobacillus amylovorus* and *Lactobacillus reuteri*; PBS, Min pigs gavaged with PBS; NC, normal control group). Comparative analysis of survival (**B**), weight loss (**C**), and diarrhea (**D**) in each group of piglets. **E** Analysis of average fluorescence intensity. **F** Comparison of histopathological changes (H&E staining) and PEDV viral load in the jejunum of piglets on day 8 post-infection. The left image is scanned at a magnification of 40×, and the right image is scanned at a magnification of 200×. **G** Histopathological scoring of tissues. The results are presented as the means ± SD, and statistical significance was calculated by one-way ANOVA. *P < 0.05; **P < 0.01; ****P < 0.0001

We developed a bacterial protection model to confirm that *Lactobacillus reuteri* and *Lactobacillus amylovorus* may play a part in the PEDV process in pigs (Fig. 5A). To determine the colonization profile of bacterial strains in the gut, we administered *Lactobacillus reuteri* and *Lactobacillus amylovorus* to piglets for three consecutive days. Analysis of the intestinal contents was conducted on the 3rd, 7th, and 14th day post-administration. The results indicated successful colonization of both bacterial strains within the gastrointestinal tracts of Min and Landrace pigs (Fig. S5G). We utilized a combination of antibiotics for microbial exhaustion to eliminate the background of mammary piglet intestine bacteria and then 16S sequencing verification to check the effectiveness of the elimination. The outcomes demonstrated that antibiotic treatment reduced the number of pig intestinal OTUs (Fig. S5A), microbial diversity (Fig. S5B, C), and species abundance (Fig. S5E, F) and changed the structure of the microbiota (Fig. S5D). Subsequently, *Lactobacillus reuteri* and *Lactobacillus amylovorus* were supplemented alone or jointly, and PEDV was administered on day 0 to observe the protective effect over the next 2 weeks. As expected, pig mortality (Fig. 5B), weight loss (Fig. 5C), and diarrhea (Fig. 5D) were improved following the addition of *Lactobacillus reuteri* and *Lactobacillus amylovorus*. Additionally, feeding *Lactobacillus reuteri* and *Lactobacillus amylovorus* helped reduce intestinal villus loss, intestinal epithelial cell necrosis, and intestinal viral load induced by PEDV (Fig. 5F). Analysis of the viral load (Fig. 5E) and epithelial cell desquamation (Fig. 5G) also revealed a similar tendency.

Moreover, under identical experimental conditions, both bacterial strains demonstrably improved the weight loss rate (Fig. S5H) and survival rate (Fig. S5I) in Landrace pigs.

### LCA and DCA in the piglet intestine are significantly enriched after PEDV infection

**Fig. 6:**
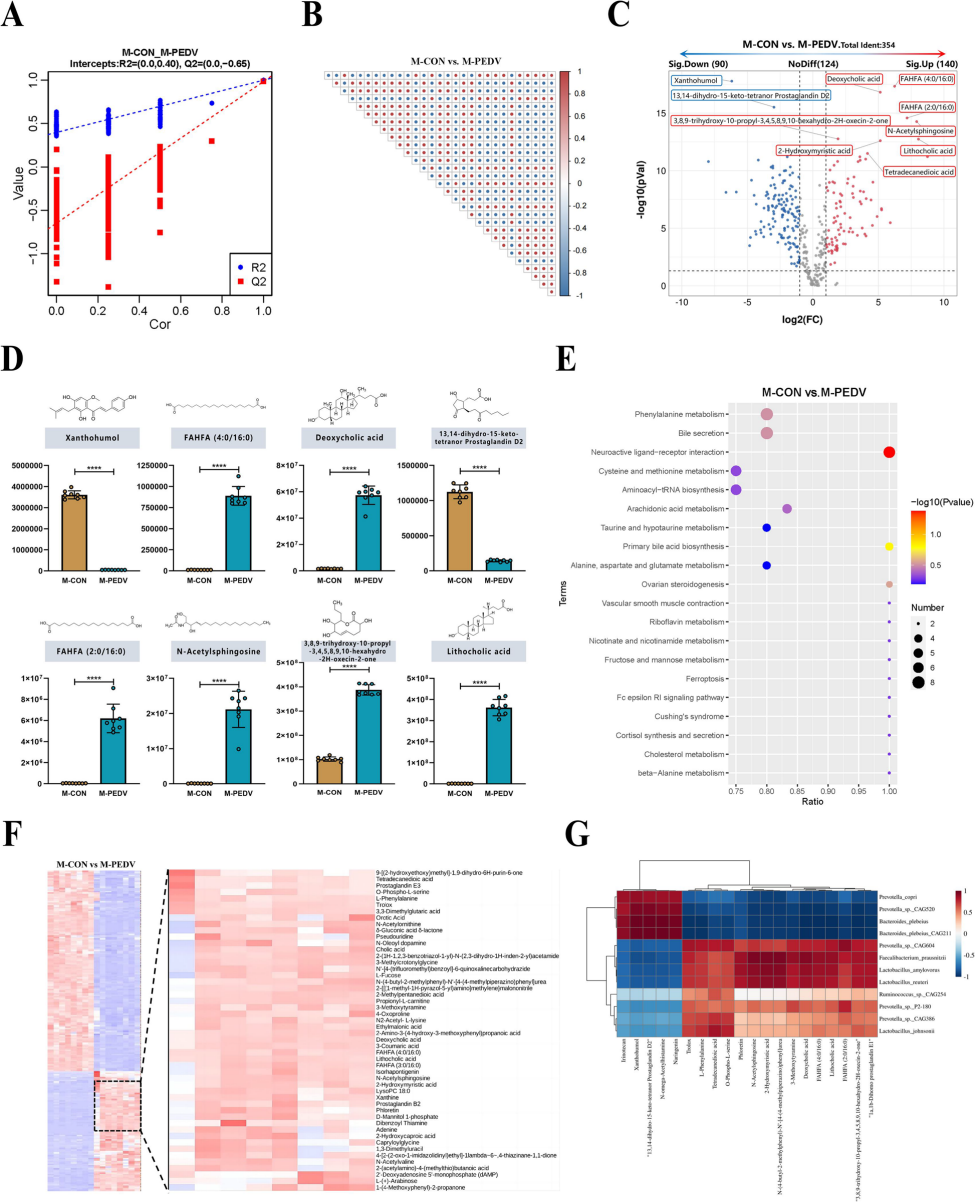
Untargeted metabolomic analysis of M-CON and M-PEDV piglet gut contents. **A** Partial least squares discrimination analysis (PLS-DA) (*n*=8/group). **B** Correlation of differentially abundant metabolites (top 30) with a significance of *P*<0.05. **C** Volcano plot analysis of differentially abundant metabolites with a threshold of VIP > 1.0, FC > 1.5, or FC < 0.667, and *P*-value < 0.05. **D** Metastats analysis counted the significantly up and downregulated differential metabolites (top 8). **E** Functional comparative analysis of metabolic components in the KEGG database (top 20). **F** Hierarchical clustering analysis of metabolic expression patterns. **G** Correlation analysis between macrogenomics and metabolomics based on Pearson correlation coefficient. The results are presented as the means ± SD, and statistical significance was calculated by one-way ANOVA. ****P < 0.0001

Next, we subjected piglet intestinal feces to nontargeted metabolomics. PLS-DA (Fig. 6A) validated the accuracy of the assay model followed by metabolite analysis and correlation between metabolites (top 30) (Fig. 6B). The PCA results showed that PEDV infection significantly changed the metabolic components of the piglet intestine (Fig. S6A). In addition, the results of the differences showed that 354 metabolites were identified, and the number of significantly upregulated and downregulated metabolites was 140 and 90, respectively (Fig. 6C). After PEDV infection, xanthohumol and 13,14-dihydro-15-keto-tetranor prostaglandin D2 in the piglets were significantly reduced, while deoxycholic acid, lithocholic acid, FAHFA (2:0/16:0), FAHFA (4:0/16:0), N-acetylsphingosine, and 3,8,9-trihydroxy-10-propyl-3,4,5,8,9,10-hexahydro-2H-oxecin-2-one increased significantly (Fig. 6D), and sample cluster analysis also showed the same results (Fig. 6F). KEGG was used to examine the metabolic component function concurrently. After PEDV infection, cysteine and methionine metabolism and aminoacyl-tRNA biosynthesis were both greatly decreased, while neuroactive ligand‒receptor interactions were significantly enriched (Fig. 6E). Next, the metabolic components that were enriched were assessed using the HMBD database. The results showed that metabolites were significantly enriched in lipids and lipid-like molecules, organic acids, and derivatives (Fig. S6B). We verified whether *Lactobacillus reuteri* and *Lactobacillus amylovorus* were correlated with the different metabolites. According to relevant analyses, there was a significant positive correlation between *Lactobacillus reuteri* and *Lactobacillus amylovorus*, deoxycholic acid, and lithocholic acid (Fig. 6G). Concurrently, analysis of the bacterial culture supernatant revealed that *Lactobacillus reuteri* and *Lactobacillus amylovorus* possess the capability to transform primary bile acids into DCA and LCA (Fig. S6D, E). Additionally, we discovered that the expression of 7-alpha-hydroxylsteroid dehydrogenase and choloylglycine hydrolase is increased in secondary bile acid production pathways (Fig. S6C).

### LCA provides protection against PEDV

**Fig. 7:**
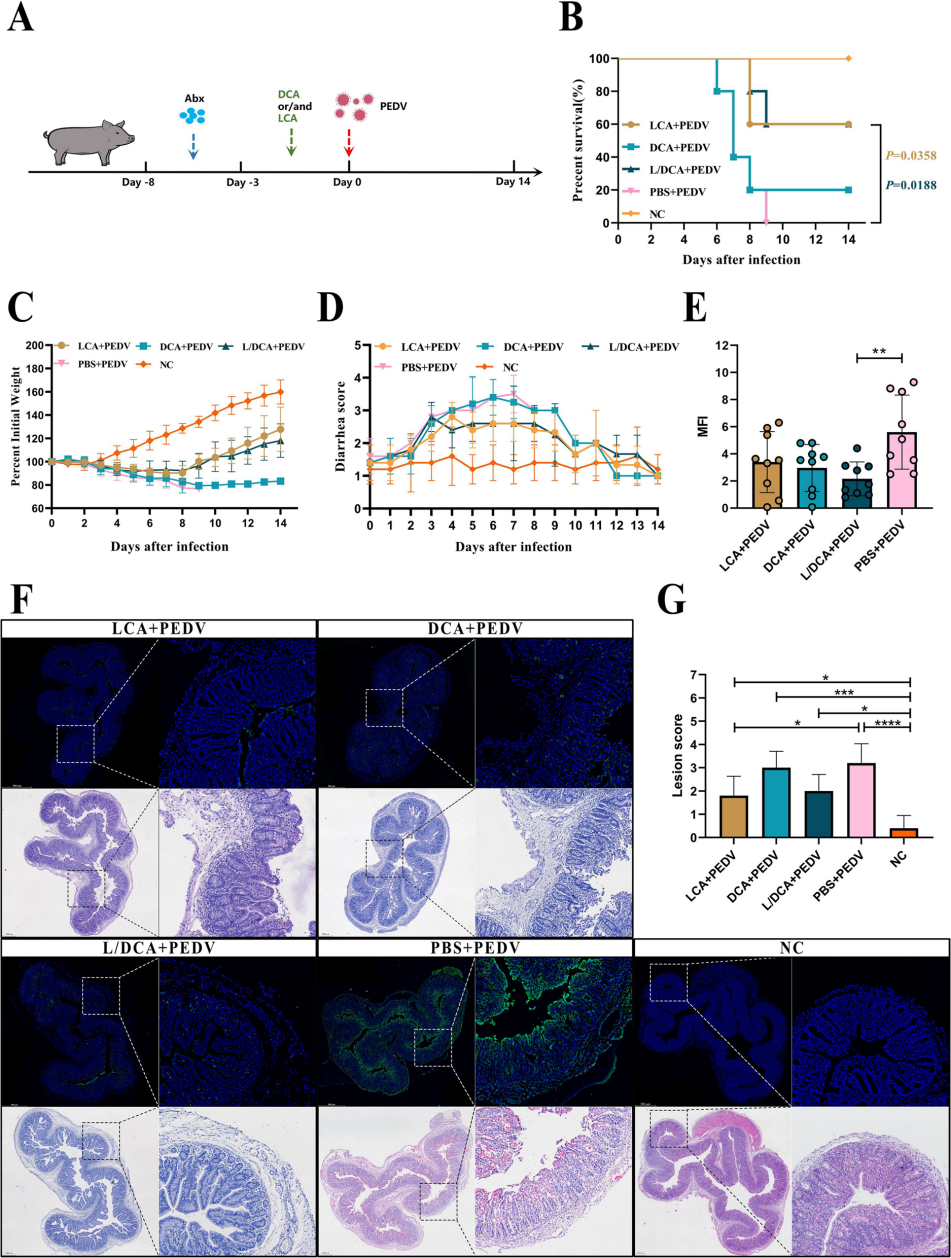
Efects of DCA and LCA on PEDV infection in piglets. **A** Protective model of DCA and LCA in piglets. Comparative analysis of survival (**B**), weight loss (**C**), and diarrhea (**D**) in each group of piglets. **E** Analysis of average fluorescence intensity. **F** Comparison of histopathological changes (H&E staining) and PEDV viral load in the jejunum of piglets on day 8 post-infection. The left image is scanned at a magnification of 40×, and the right image is scanned at a magnification of 200×. **G** Histopathological scoring of tissues. The results are presented as the means ± SD, and statistical significance was calculated by one-way ANOVA. *P < 0.05; **P < 0.01; ***P < 0.001; ****P < 0.0001

Bile acid is decomposed into secondary bile acid in the intestine, and secondary bile acid has biological activity. For example, UCDC can inhibit the invasion of SARS-CoV-2 to prevent virus infection [37]. Therefore, we speculate that DCA and LCA, which are secondary bile acids, may contribute to PEDV infection. We established a supplementary bile acid protection model to confirm this presumption (Fig. 7A). Similarly, we employed a combination of antibiotics for microbial exhaustion and 16S sequencing to verify the exhaustion efficiency. As before, antibiotic treatment reduced the number of pig intestinal OTUs (Fig. S7A), microbial diversity (Fig. S7B, C), and species abundance (Fig. S7E, F) and changed the structure of the microbiota (Fig. S7D). Subsequently, DCA and LCA were supplemented alone or jointly, and PEDV was administered on day 0 to observe the protective effect over the next 2 weeks. Surprisingly, only in the group supplemented with LCA did the survival (Fig. 7B) and weight loss (Fig. 7C) of piglets improve. At the same time, diarrhea (Fig. 7D) was also relieved accordingly. Furthermore, compared to the infected group, the supplementation of LCA resulted in reduced intestinal damage in piglets, while the piglets supplemented with LCA and DCA exhibited decreased viral load in the intestines (Fig. 7F). This phenomenon was also supported by the findings of epithelial cell shedding (Fig. 7G) and PEDV fluorescence intensity (Fig. 7E). In addition, LCA also enhanced the weight loss rate (Fig. S7G) and survival rate (Fig. S7H) in Landrace pigs.

### LCA enhanced the intestinal infiltration of CTLs

To investigate how LCA functions, we examined the expression of PEDV-specific IgA in the duodenum (Fig. S8A), jejunum (Fig. S8C), and ileum (Fig. S8D) of piglets. Disappointingly, LCA did not enhance IgA expression. In addition, dendritic cells and macrophages can process and clear viruses and scale down the viral infection cycle to restore the body to its normal state. Next, we analyzed whether LCA affected dendritic cells and macrophages in the small intestine and mesenteric lymph nodes of piglets. Analysis was performed by circling dendritic cells and macrophages after gating (Fig. S8B). The results showed that LCA treatment did not affect the changes in the number of dendritic cells (Fig. S8E, F) and macrophages (Fig. S8G, H) in the small intestine and mesenteric lymph nodes of piglets, suggesting that LCA may affect disease recovery through other immune pathways.

**Fig. 8:**
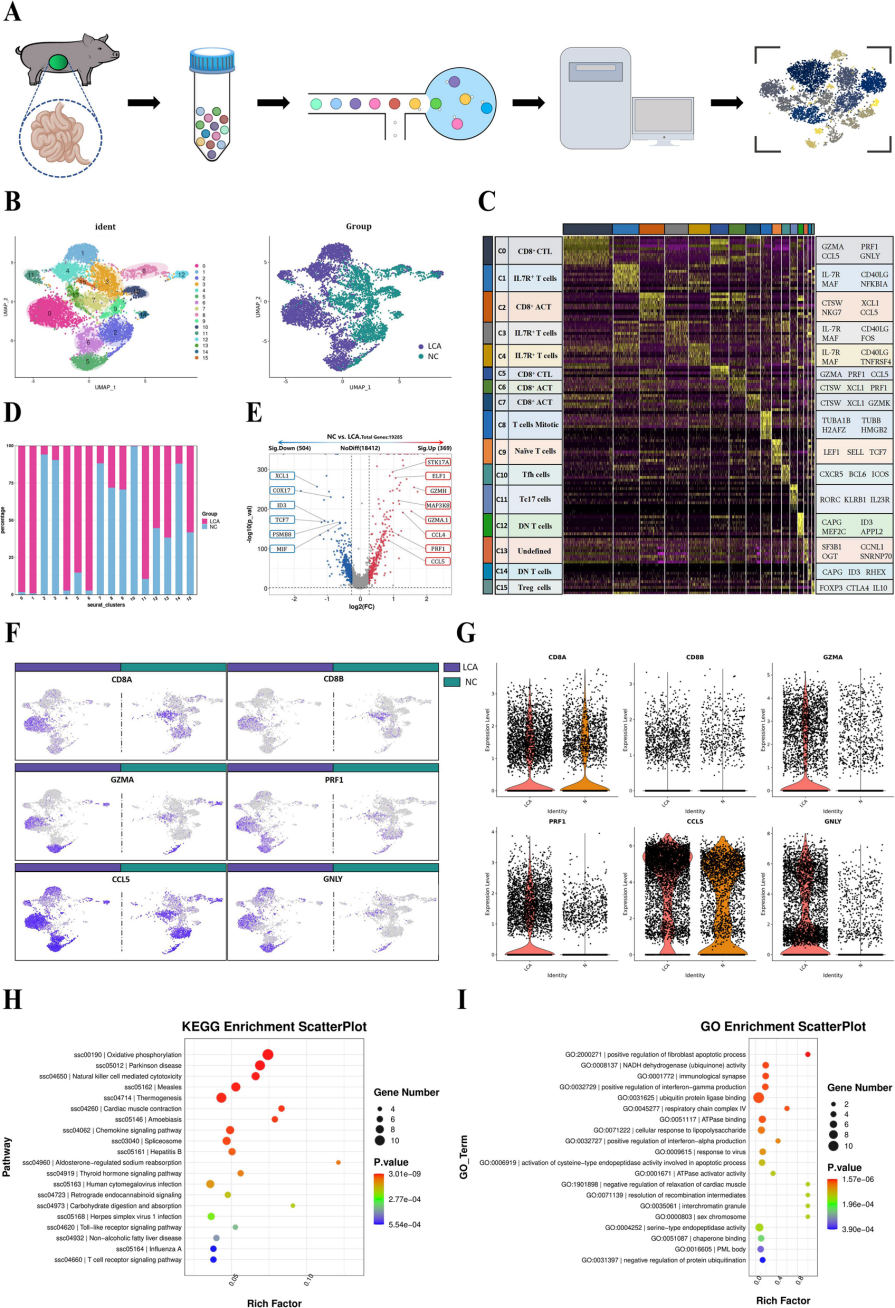
Single-cell RNA sequencing analysis of piglet intestines. **A** Workflow for single-cell and RNA-seq of isolated T cells from pigs. **B** Dimensionality reduction analysis based on the UMAP algorithm, with the number of clusters corresponding to those listed in **C**. **C** Heatmap clustering data from single-cell analysis of LCA-treated or untreated groups, where the cell clusters are indicated by the upper color block. The signature genes that were differentially expressed in each cluster are shown on the far right, and the names of the subgroups of cell clusters are shown on the far left. **D** Percentage of cells in the LCA-treated or untreated group in the cluster. **E** Volcano plot for differential genetic screening of cells in the LCA-treated or untreated groups. Analysis of the expression of the marker genes CD8A, CD8B, GZMA, PRF1, CCL5, and GNLY in cells from LCA-treated or untreated groups by UMAP (**F**) and cytogenetic enrichment (**G**) methods. Differential gene function analysis of the CD8^+^ CTL cluster in the KEGG (**H**) and GO (**I**) databases

Subsequently, we hypothesized that T cells might play a major role after LCA supplementation, and we performed single-cell transcriptome analysis by microbead isolation of CD3^+^ T cells from the piglet intestine (Fig. 8A). The expression matrix data of cells are dropped onto a two-dimensional plane by dimensionality reduction, and the data are distinguished by similarity to obtain subgroup statistics of cells, which are used for accurate differentiation of cell types and subtypes and their visualization. After filtering to obtain high-quality cells, the algorithm homogenizes the expression matrix, downscales the data, and bins the cells into groups (clusters). A high-quality scRNA-seq dataset consisting of 7847 cells describing the groups from either the supplemented or unsupplemented LCA groups post-challenge was generated.

T-cell clusters were visualized using UMAP (uniform manifold approximation and projection). We identified 16 unique clusters based on gene expression profiles (Fig. 8B). Cluster analysis of 10 major cell types include the CD8^+^ CTL cell population with high expression of cytotoxic genes GZMA, PRF1, and CCL5; IL-7R^+^ T-cell population with high expression of IL-7R, CD40LG, and MAF (including CD4/8 expression); CD8^+^ ACT cell population with expression of the cell activation-related genes CTSW and XCL1; mitotic T-cell population with expression of the mitosis-related genes TUBA1B, TUBB, H2AFZ, and HMGB2 [38]; naïve T cells with expression of LEF1, SELL, and TCF7; TFH cell population expressing CXCR5, BCL6, and ICOS; CD8 T cells expressing RORC, KLRB1, and IL23R; a tc17 cell population expressing CD8, RORC, KLRB1, and IL23R; a double-negative (DN) T-cell population not expressing CD4/8; Treg cells expressing the Treg-related genes FOXP3, CTLA4, and IL10 cell clusters; and a few unspecified clusters (Fig. 8C). In addition, the proportion of different cell populations among the samples was determined to characterize the distribution of LCA-affected T cells (Fig. 8D).

To investigate whether LCA affects the function of T cells, the two groups were screened for differentially expressed genes, and the results showed that a total of 19,285 genes were screened, of which the number of significantly upregulated genes was 369 and the number of significantly downregulated genes was 504. Compared with the control group, LCA supplementation significantly elevated the expression of genes related to cytotoxic effects in the intestine, such as GZMH, GZMA.1, CCL5, and PRF1. Furthermore, genes such as XCL1, COX17, ID3, and TCF7 were significantly downregulated (Fig. 8E). In addition, enrichment visualization and statistics of the two groups for CTL-related genes, namely, CD8A/B, GZMA, PRF1, CCL5, and GNLY, showed that cells expressing CD8^+^ CTL-related genes were significantly enriched in the LCA supplemented group (Fig. 8F, G). To resolve the possible functions of LCA regulation of CD8^+^ CTLs, functional prediction analysis was performed for CD8^+^ CTLs population in the KEGG and GO databases. KEGG predictions showed functional enrichment of CD8^+^ CTL population in the functional categories of oxidative phosphorylation, Parkinson’s disease, and natural killer cell-mediated cytotoxicity (Fig. 8H). Moreover, the results of the GO database comparison showed that the CD8^+^ CTL population had higher enrichment in positive regulation of fibroblast apoptotic processes and respiratory chain complex IV, while it was enriched in ubiquitin protein ligase binding function and was enriched in a higher number of genes (Fig. 8I).

### LCA enhances intestinal SLA-I expression and subsequently recruits more CTLs via FXR

**Fig. 9:**
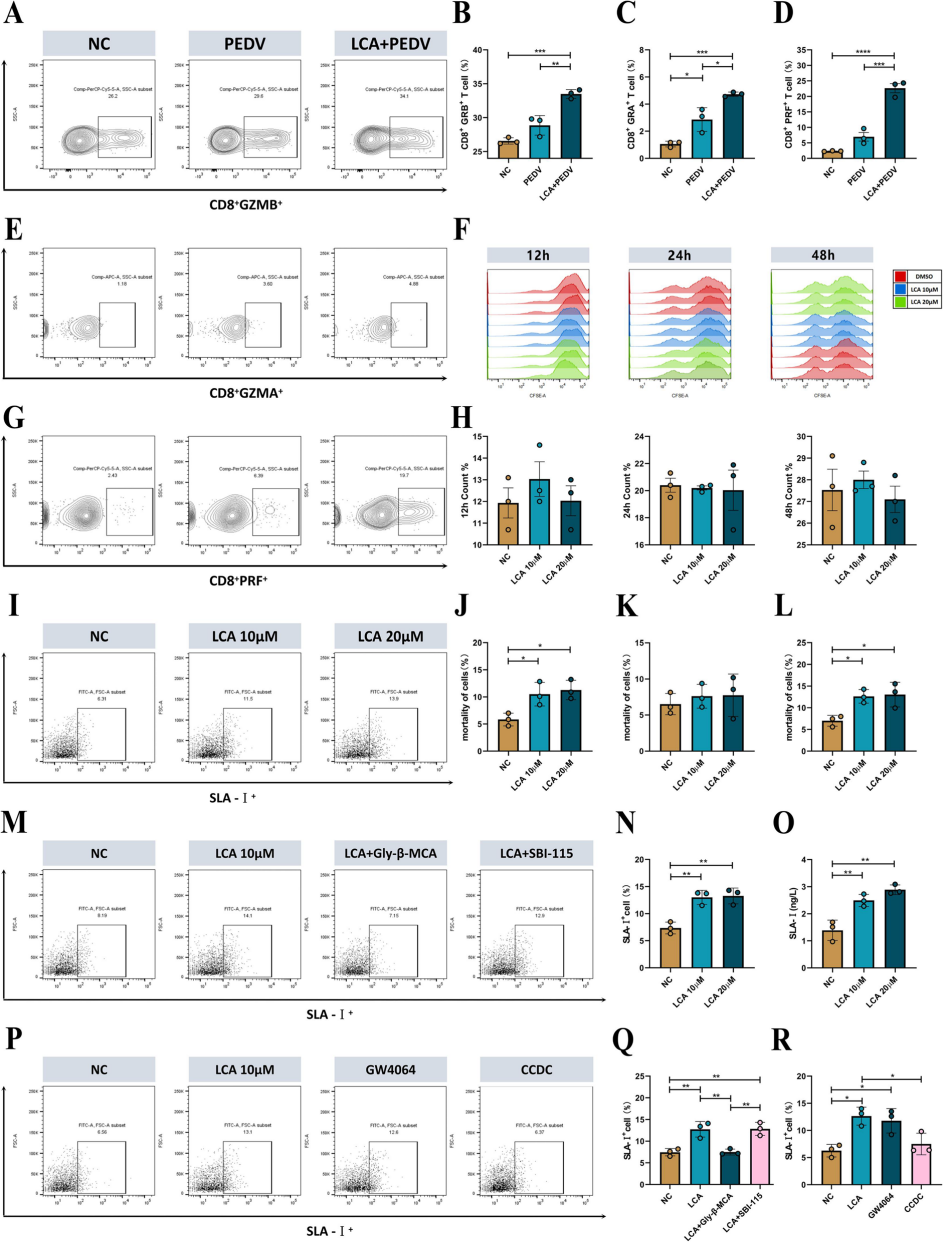
Mechanistic analysis of the protective effect of LCA. GZMB (**A**), GZMA (**E**), and PRF (**G**) expression in CD3^+^CD8^+^ T cells was measured by flow cytometry on day 8 after PEDV infection in piglets, and the proportions of GZMB (**B**), GZMA (**C**), and PRF (**D**) cells were statistically analyzed (*n*=3). **F** Cell proliferation assay of CD8^+^ cells treated with 10/20 μM LCA and anti-CD3/28 for 12/24/48 h using CFSE labeling (*n*=3). **H** Cell proliferation ratio of CD8^+^ cells at 12/24/48 h (*n*=3). **I** SLA-I expression was analyzed by flow cytometry after 10/20 μM LCA treatment of IPEC-J2 cells for 12 h and was statistically analyzed (**N**) (*n*=3). **O** IPEC-J2 cells were treated with 10/20 μM LCA for 12 h before cell lysis and subsequent ELISA for SLA-I expression (*n*=3). **M** The expression of SLA-I was analyzed by flow cytometry in the presence and absence of SBI-115 and Gly-β-MCA in IPEC-J2 cells treated with 20 μM LCA for 12 h, and was statistically analyzed (Q) (*n*=3). **P** SLA-I expression was analyzed by flow cytometry in the presence and absence of CCDC and GW4064 in IPEC-J2 cells treated with 20 μM LCA for 12 h, and was statistically analyzed (**R**) (*n*=3). The results are presented as the means ± SD, and statistical significance was calculated by one-way ANOVA. *P < 0.05; **P < 0.01; ***P < 0.001; ****P < 0.0001

Next, we verified whether LCA supplementation enriched CTLs in the intestine by flow cytometry, and the circling gate strategy is shown in Fig. S9A. The results were consistent with the sequencing results, and LCA supplementation elevated the number of CD8^+^GRB^+^ T cells (Fig. 9A, B), CD8^+^GRA^+^ T cells (Fig. 9E, C), and CD8^+^PRF^+^ T cells (Fig. 9G, D) in the intestine of piglets compared with the untreated group. To determine whether LCA acts directly on CTL enrichment and the expression of associated effectors, we isolated CD8^+^ T cells from the intestine of healthy piglets, stimulated and activated them in vitro by anti-CD3/CD28, and treated them with LCA. The results showed that LCA did not act directly on CTL amplification at 12 h, 24 h, and 48 h (Fig. 9F, H). Furthermore, there was no significant difference in the expression of the CD8^+^ CTL intracellular effectors GRB (Fig. S9B, C), GRA (Fig. S9D, F), and PRF (Fig. S9E, K) at 24 h. In addition, IL-2 is essential for initial T-cell proliferation and enhances T-cell enhancement and survival. Therefore, we isolated CD4^+^ and CD8^+^ cells by the magnetic bead method and examined IL-2 expression levels after the same stimulation treatment, which showed that LCA treatment failed to enhance IL-2 expression in CD4^+^ (Fig. S9G) and CD8^+^ (Fig. S9H) cells, suggesting that LCA enhanced the cytotoxic effect of CTLs by other means.

Since CTLs perform killing functions with MHC-1 restriction, we stimulated IPECJ-2 cells in vitro with different concentrations of LCA. Surprisingly, the results of flow cytometry (Fig. 9I, N) and ELISA (Fig. 9O) showed that LCA treatment increased SLA-I expression in IPECJ-2 cells. Next, we wanted to identify the key receptors for LCA action on IPECJ-2 cells. Previous studies have demonstrated that TGR5 and FXR are the major receptors for bile acids, so we treated the cells with the TGR5 and FXR receptor inhibitors SBI-115 and Gly-β-MCA and treated them with LCA. The results of flow cytometry (Fig. 9M, Q) and ELISA (Fig. S9J) showed that the addition of the FXR inhibitor blocked the high expression of SLA-I in IPECJ-2 cells, while the TGR5 inhibitor had no blocking effect. Moreover, we treated IPECJ-2 cells with the receptor agonists CCDC and GW4064 of TGR5 and FXR, and the results of flow cytometry (Fig. 9P, R) and ELISA (Fig. S9I) showed that the FXR receptor agonists increased SLA-I expression in IPECJ-2 cells, and the above results indicated that LCA elevated SLA-I expression through FXR. Furthermore, to demonstrate the relationship between the high expression of SLA-I and the killing function of CTLs, we isolated total CD8^+^ T cells from the intestine of PEDV-infected piglets while using epitope peptides to encapsulate IPECJ-2 cells in vitro and treated them with LCA under different conditions and showed that LCA treatment increased the killing efficiency of CTLs (Fig. 9J). Furthermore, treatment with Gly-β-MCA eliminated the additional killing efficiency of CTLs (Fig. 9K), while SBI-115 had no effect (Fig. 9L).

## Discussion

**Fig. 10:**
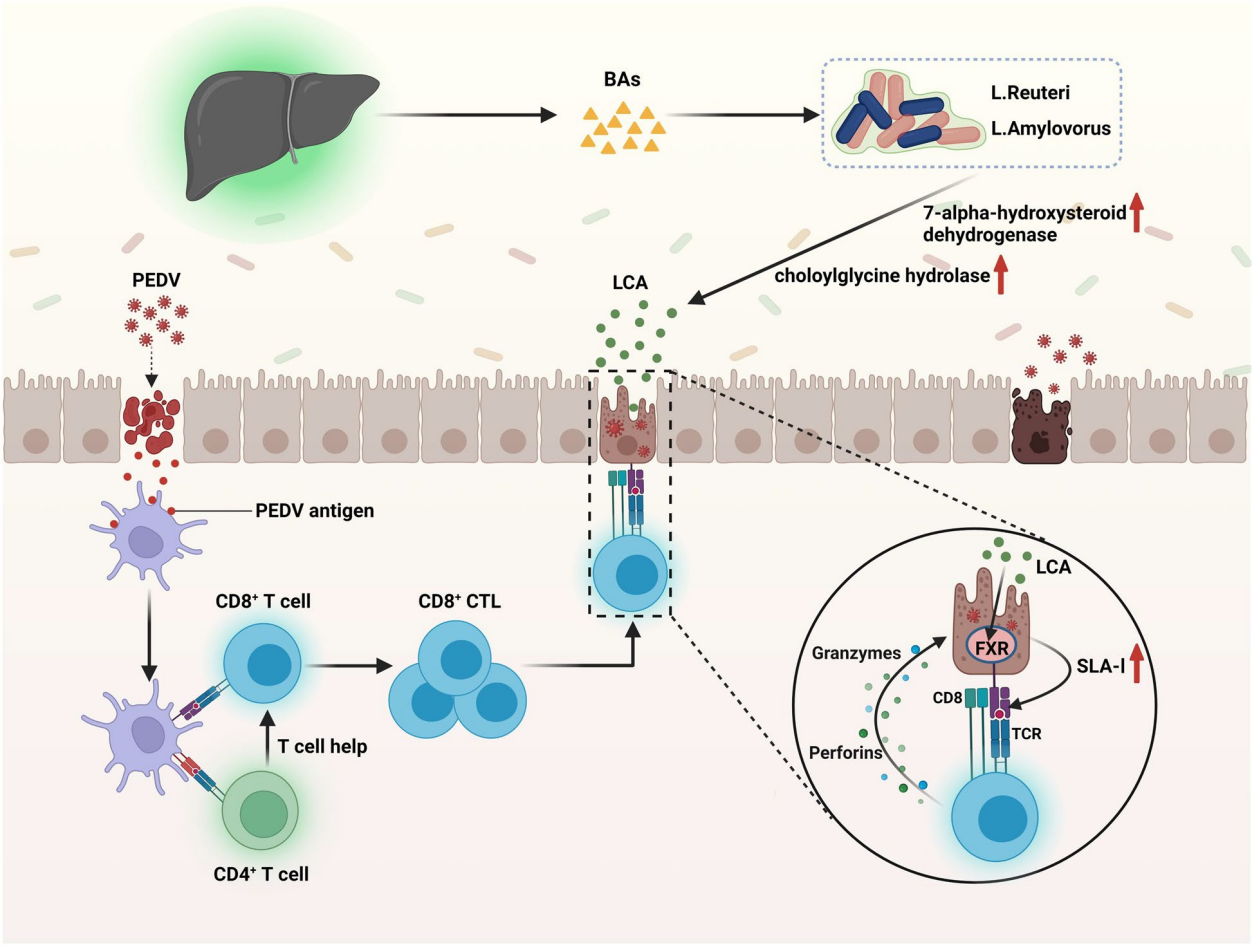
Mechanisms of PEDV infection alleviation by the gut microbes. Bile acids are produced in the liver and enter the intestine via the digestive system. Once in the intestine, primary bile acids may be broken down into LCA by the bacteria *Lactobacillus amylovorus* and *Lactobacillus reuteri* by increasing their expression of 7-alpha-hydroxylsteroid dehydrogenase and choloylglycine hydrolase to thus catabolize bile acids to LCA. LCA increases SLA-I expression in porcine intestinal epithelial cells via FXR, which allows accelerated recruitment of CD8^+^ CTLs to PEDV-infected target cells, followed by efficient clearance of target cells by releasing killing factors such as granzyme and perforin, thereby alleviating PEDV infection. The mechanisms diagram was created using BioRender.com

This study provides evidence that the differential gut microbiota of Min pigs and Landrace pigs determines the difference in severity of PEDV infection. The intestinal microbiota mitigated PEDV infection through the mechanisms shown in Fig. 10. PEDV infection in Min pigs enriched *Lactobacillus reuteri* and *Lactobacillus amylovorus* and produced more LCA, which increased SLA-I expression in intestinal epithelial cells through the FXR receptor pathway, thereby recruiting more effector CTLs and efficiently removing target cells to accelerate disease regression.

The diversity of gut microbes increases the intestinal immune memory pool, the intestinal immune system and gut microbes provide checks and balances, and some bacteria control the growth and evolution of the intestinal immune system [39]. Studies have shown that different strains of animals have different states of host receptivity to pathogen invasion. Differences in innate immune responses influence the susceptibility of different strains of chickens to NDV infection [40]. In our study, significant differences in infection were also shown after PEDV challenge in Min pigs and Landrace pigs under the same conditions. FMT has accelerated the discovery of gut microbial functions. FMT was found to reduce diarrhea in preweaned calves, to regulate the intestinal flora, and possibly to enhance growth performance in one study [41]. Additionally, extensive research on FMT has been performed with a number of metabolic illnesses, including diabetes and obesity. Studies have shown that FMT from lean donors significantly improves insulin sensitivity in obese subjects with metabolic syndrome [42]. The FMT method was applied in our research to validate the potential contribution of gut microorganisms to viral infections and to screen for disease-specific bacterial indicators and important target molecules among the gut microbes.

Previous studies analyzing the gut microbial composition of piglets infected with PEDV differed from our findings in that the abundance of *Lactobacillus* decreased after infection with PEDV [43]. Another study showed a reduction in *Bacteroidota* abundance and a significant increase in the *Firmicutes* and Lactobacillus abundances in the gut of piglets following PEDV infection, which is consistent with our findings [44]. We found an interesting phenomenon in which the number of intestinal OTUs was significantly lower in Min pigs than in Landrace pigs. However, the number of intestinal OTUs in Min pigs increased, and the number of intestinal OTUs in Landrace pigs decreased after infection with PEDV. It appears that the greater abundance of some possible bacteria influences the process of viral infection, and this effect may be direct or indirect based on the lower viral susceptibility of Min pigs. Recent studies have demonstrated differences in the gut microbial composition of Lu Chuan (LC) piglets and Landrace pigs, with a reduction in the relative abundance of Shigella in the gut of LC piglets following PEDV infection and differences in the species and number of dominant phyla and genera in the same intestinal segment [45]. This study revealed differences in the microbiota of Min pigs and Landrace pigs under PEDV infection by α and β diversity. The invasion of the gut by PEDV causes an imbalance in the existing gut microbes and enhances the risk of infection by other conditioned pathogenic microorganisms.

A growing corpus of research has demonstrated that the distinctive strains among the gut microbiota are frequently essential for disease prevention. Mice with unilateral ureteral obstruction (UUO) and CKD had lower levels of *Bacteroides fragilis* in their feces, but supplementation with *Bacteroides fragilis* reduced renal fibrosis in mice with UUO and adenosine models [8]. More endogenous *Bifidobacterium animalis* is found in the guts of mice that can survive a deadly influenza attack, proving a relationship between the influenza virus and the diverse responses of the gut microbiota [46]. The abundance of *Lactobacillus reuteri* and *Lactobacillus amylovorus* significantly increased in the Min pigs subjected to PEDV challenge in the current study, which may be a major factor in the anti-infective effect of Min pigs and was confirmed in subsequent experiments. The therapeutic potential of *Lactobacillus reuteri* in treating acute viral diarrhea in children has been demonstrated in clinical trials using *Lactobacillus reuteri* as a therapeutic agent for acute rotavirus diarrhea in children [47]. *Lactobacillus reuteri* administration results in gastrointestinal colonization and significantly reduces diarrhea in children. In addition, *Lactobacillus reuteri* regulates immune cells, and its surface and secreted metabolites support dendritic cell maturation, IL-10 production, and inflammation suppression, which may be a key mechanism for preserving immunological homeostasis in the gut [48]. In vitro tests revealed that *Lactobacillus amylovorus* AA099 had the strongest antiviral activity against echoviruses in a study of probiotics against enteroviruses [49]. In this study, the combination of *Lactobacillus reuteri* and *Lactobacillus amylovorus* proved to be more effective than either strain supplemented alone, indicating that these two strains may interact to combat PEDV.

The gut microbes can indirectly regulate disease development and immune responses through their own or derived metabolites, depending on the metabolites and their interaction with receptors on host cells, and these receptors can in turn activate or inhibit signaling pathways that can be beneficial or detrimental to the health of the host [50]. A recent study showed that the use of ursodeoxycholic acid (UDCA) to reduce FXR signaling and to downregulate ACE2 in human lung, bile duct cells, and intestinal-like organs, as well as in corresponding tissues in mice, reduced the susceptibility of human lung and liver to SARS-CoV-2 infection in vitro, in vivo, and by ectopic perfusion [37]. PEDV, which is the same coronavirus as SARS-CoV-2, significantly elevated the secondary bile acid intestinal metabolites DCA and LCA after infecting the intestine of Min pigs, and subsequent experiments confirmed the unique protective efficacy of LCA. Furthermore, it has been shown that LCA acts through the GPCR-IFN-λ3-ISG15 signaling axis in IPEC-J2 cells to inhibit PDCoV replication [51]. We have therefore identified the secondary bile acid intestinal metabolite LCA as the next target for studying the mechanism of action.

The host gut immune system and gut microbes have interacted reciprocally during a long period of coevolution. The constant colonization of the body by microbes causes the recruitment of various immune cell types from birth [52]. Gut mucosal immunity, particularly SIgA, is vital for defending against intestinal pathogens. In addition to killing target cells indirectly through ADCC (antibody-dependent cell-mediated cytotoxicity) activity, it also neutralizes the virus and stops viral invasion [53]. Unfortunately, LCA did not operate in the current investigation to increase IgA and influence the expression of macrophages and DCs in myeloid cells. When PEDV invades the intestinal epithelium, the APC catches the virus antigen and delivers it to the initial T cells. The initial T cells then differentiate into effector T cells, which are able to identify and destroy pathogen-infected cells. Therefore, we hypothesize that LCA may play a role in the T-cell response process. In this work, the distribution of CD3^+^ T cells in the swine gut was evaluated by single-cell sequencing, and 16 cell types were classified. CD8^+^ CTLs were highly enriched in the LCA-treated group, and cytotoxicity-related genes such GZMH, GZMA.1, CCL5, and PRF1 were significantly increased. This is consistent with our hypothesis that LCA may ultimately regulate the process of viral infection through T-cell action. Additionally, by regulating T-cell activation and metabolism, bile acids play a crucial role in the battle against HBV infection. Targeted modulation of bile acids may be a useful therapeutic approach for host virus defense [54]. Since metabolites acted directly on immune cells in previous studies, we hypothesized that LCA also directly affects CD8^+^ T-cell expression. Surprisingly, the in vivo findings matched the single-cell sequencing data, whereas coculture of LCA and in vitro activated CD8^+^ T cells failed to result in CD8^+^ T-cell growth or the production of the intracellular factors GZMA, GZMB, and PRF in effector CTLs. This result suggests that LCA may regulate CD8^+^ T cells through an indirect pathway. In mice with colorectal cancer (CRC), all-trans retinoic acid (AtRA) has been found to suppress tumorigenesis and to regulate the activation of cytotoxic CD8^+^ T lymphocytes by upregulating tumor cell MHC-I to slow tumor growth [55]. In addition, rotavirus may also inhibit MHC-I expression in vivo, which may limit T-cell killing of rotavirus-infected enterocytes [56]. Therefore, we investigated the effect of LCA on SLA-I expression in pig intestinal epithelial cells. As expected, LCA increased SLA-I expression in porcine intestinal epithelial cells, and subsequent screening with receptor inhibitors and agonists showed that FXR was the primary receptor for LCA function on porcine intestinal epithelial cells, which was confirmed by CTL killing assays.

In conclusion, our results suggest that the variability of PEDV infections in Min pigs is mediated by the gut microbes. More notably, LCA derived from *Lactobacillus reuteri* and *Lactobacillus amylovorus* provides defense. LCA enhanced SLA-I expression in pig intestinal epithelial cells through FXR receptors, which in turn attracted more CD8^+^ CTLs to fight PEDV infection. Our current findings further demonstrate the role of the gut microbiota in regulating host gut health through metabolites.

### Supplementary Information

**Additional file 1:** **Fig. S1.** Time charts for animal experiments A, B, C, and D. **Fig. S2.** Genus co-occurrence network and evolutionary tree of intestinal microorganisms in Min pigs and Landrace pigs. (A) The association interactions of the top 50 genera were analysed by calculating correlation indices for all samples. (B) Phylogenetic tree constructed from representative sequences of species at the genus level (top 100). **Fig. S3.** Validation of the depletion of gut microbes with combination antibiotic treatment. (A) Statistics on the number of OTUs of gut microbes in each group. (B) Chao1 estimate of the total number of species contained in the community samples. (C) Shannon’s assessment of the total number of taxa in the sample and their proportions. (D) PCoA based on weighted UniFrac distances to determine changes in microbial structure. The top 10 species at the phylum (E) and genus (F) levels were calculated to assess the species with the highest abundance and their proportions (*n*=3). **Fig. S4.** Macrogenomic analysis of the gut microbes of M-CON and M-PEDV. (A) Clustering analysis of species among the M-CON and M-PEDV gut microbes. (B) Functional enrichment from the KEGG database at six levels. **Fig. S5.** Intestinal microbial depletion and analysis of bacterial colonization and protective capabilities. (A) Statistics on the number of OTUs of gut microbes in each group. (B) Chao1 estimate of the total number of species contained in the community samples. (C) Shannon’s assessment of the total number of taxa in the sample and their proportions. (D) PCoA based on weighted UniFrac distances to determine changes in microbial structure. The top 10 species at the phylum (E) and genus (F) levels were calculated to assess the species with the highest abundance and their proportions (*n*=3). (G) Analysis of Colonization Potentials of *Lactobacillus reuteri *and *Lactobacillus amylovorus*. (H) Weight Loss Rates in Landrace pigs. (I) Survival Rate Analysis in Landrace pigs. **Fig. S5.** Intestinal microbial depletion and analysis of bacterial colonization and protective capabilities. (A) Statistics on the number of OTUs of gut microbes in each group. (B) Chao1 estimate of the total number of species contained in the community samples. (C) Shannon’s assessment of the total number of taxa in the sample and their proportions. (D) PCoA based on weighted UniFrac distances to determine changes in microbial structure. The top 10 species at the phylum (E) and genus (F) levels were calculated to assess the species with the highest abundance and their proportions (*n*=3). (G) Analysis of Colonization Potentials of *Lactobacillus reuteri *and *Lactobacillus amylovorus*. (H) Weight Loss Rates in Landrace pigs. (I) Survival Rate Analysis in Landrace pigs. **Fig. S6.** LC‒MS/MS analysis of the M-CON and M-PEDV group faeces. (A) PCA of the intestinal metabolites of the M-CON and M-PEDV groups. (B) Annotation of the Human Metabolome Database (HMDB) of intestinal metabolites. (C) KEGG functional validation of differentially expressed genes in the secondary bile acid synthesis pathway. Qualitative (D) and quantitative (E) Detection of DCA and LCA in the Supernatants of *Lactobacillus reuteri *and *Lactobacillus amylovorus*. **Fig. S7.** Analysis of intestinal microbial depletion and strain protective ability. (A) Statistics on the number of OTUs of gut microbes in each group. (B) Chao1 estimates of the total number of species contained in the community samples. (C) Shannon’s assessment of the total number of taxa in the sample and their proportions. (D) PCoA based on weighted UniFrac distances to determine changes in microbial structure. The top 10 species at the phylum (E) and genus (F)  levels were calculated to assess the species with the highest abundance and their proportions (*n*=3). (G) Weight Loss Rates in Landrace pigs. (H) Survival Rate Analysis in Landrace pigs. **Fig. S8.** Effect of LCA on intestinal immune function. IgA expression in the duodenum (A), jejunum (C) and ileum (D) of LCA-treated and untreated piglets after PEDV infection. (*n*=3/group). (B) Gating strategies for porcine dendritic cells and macrophages. Flow cytometry analysis of dendritic cell (F) and macrophage (H) expression in piglet jejunum and dendritic cell (E) and macrophage (G) expression in porcine mesenteric lymph nodes. (*n*=3/group). The results are presented as the means ± SDs, and statistical significance was calculated by one-way ANOVA. *P< 0.05; **P < 0.01. **Fig. S9.** LCA increases SLA-I expression in porcine intestinal epithelial cells via FXR and thus enhances the killing efficiency of CTLs. (A) Flow cytometry strategy to detect the expression of GZMB, GZMA and PRF in CD3+CD8+ T cells. The expression of GZMB (B), GZMA (F) and PRF (K) was detected by flow cytometry after 10/20 μM LCA and anti-CD3/28 treatment of CD8+cells for 24 h, and the percentages of cells with GZMB (C), GZMA (D) and PRF (E) were counted (*n*=5). Cells treated with 20 μM LCA in concert with anti-CD3/CD28 were isolated from CD4+ (G) and CD8+ (H) cells by magnetic beads, and the cell supernatants were analysed for IL-2 by ELISA. (J) The expression of SLA-I was detected by ELISA in the presence and absence of SBI-115 andGly-β-MCA in IPEC-J2 cells treated with 20 μM LCA for 12 h (*n*=3). (I) SLA-I expression was measured by ELISA in the presence and absence of AOB2659(CCDC) and GW4064 in IPEC-J2 cells treated with 20 μM LCA for 12 h (*n*=3). The results are presented as the means ± SDs, and statistical significance was calculated by one-way ANOVA. *P< 0.05.

## Data Availability

Sequencing data of 16S rDNA from Min pigs and Landrace pigs, sequencing data of the macrogenome, sequencing data of 16S rDNA from piglets treated with antibiotics in the FMT experiment, sequencing data of piglets treated with antibiotics before feeding *Lactobacillus reuteri* and *Lactobacillus amylovorus*, sequencing data of piglets treated with antibiotics before feeding DCA and LCA, and single-cell sequencing data have been uploaded to the NCBI Sequence Read Archive database with registration numbers PRJNA923293, PRJNA923758, PRJNA923757, PRJNA923754, PRJNA925821, and PRJNA925792, respectively. All data supporting the results of this study can be found in the manuscript or in the supplementary information. Source data are provided in this paper.
