# Supplementary material for: Gut microbiota-derived LCA mediates the protective effect of PEDV infection in piglets
Source: Microbiome. 2024 Feb 5;12:20. doi: 10.1186/s40168-023-01734-4 (PMC10840300; doi:10.1186/s40168-023-01734-4)
Supplement: Supplementary file 2 — Additional file 1: Fig. S1. Time charts for animal experiments A, B, C, and D. Fig. S2. Genus co-occurrence network and evolutionary tree of intestinal microorganisms in Min pigs and Landrace pigs. (A) The association interactions of the top 50 genera were analysed by calculating correlation indices for all samples. (B) Phylogenetic tree constructed from representative sequences of species at the genus level (top 100). Fig. S3. Validation of the depletion of gut microbes with combination antibiotic treatment. (A) Statistics on the number of OTUs of gut microbes in each group. (B) Chao1 estimate of the total number of species contained in the community samples. (C) Shannon’s assessment of the total number of taxa in the sample and their proportions. (D) PCoA based on weighted UniFrac distances to determine changes in microbial structure. The top 10 species at the phylum (E) and genus (F) levels were calculated to assess the species with the highest abundance and their proportions (n=3). Fig. S4. Macrogenomic analysis of the gut microbes of M-CON and M-PEDV. (A) Clustering analysis of species among the M-CON and M-PEDV gut microbes. (B) Functional enrichment from the KEGG database at six levels. Fig. S5. Intestinal microbial depletion and analysis of bacterial colonization and protective capabilities. (A) Statistics on the number of OTUs of gut microbes in each group. (B) Chao1 estimate of the total number of species contained in the community samples. (C) Shannon’s assessment of the total number of taxa in the sample and their proportions. (D) PCoA based on weighted UniFrac distances to determine changes in microbial structure. The top 10 species at the phylum (E) and genus (F) levels were calculated to assess the species with the highest abundance and their proportions (n=3). (G) Analysis of Colonization Potentials of Lactobacillus reuteri and Lactobacillus amylovorus. (H) Weight Loss Rates in Landrace pigs. (I) Survival Rate Analysis in Landrace pigs. Fig. S5. Inte [file 40168_2023_1734_MOESM1_ESM.pdf]

Gut microbiota-derived LCA mediates the protective effect of PEDV infection in piglets

Jun-Hong Xing [1], Tian-Ming Niu [1], Bo-Shi Zou [1], Gui-Lian Yang [1], Chun-Wei Shi [1], Qing-Song Yan [1], Ming-Jie Sun [1], Tong Yu [1], Shu-Min Zhang [1], Xi-Ze Feng [1], Shu-Hui Fan [1], Hai-Bin Huang [1], Jun-Hong Wang [1], Ming-Han Li [1], Yan-Long Jiang [1], Jian-Zhong Wang [1], Xin Cao [1], Nan Wang [1], Yan Zeng [1], Jing-Tao Hu [1], Di Zhang [1], Wu-Sheng Sun [1], Wen-Tao Yang [1,*], Chun-Feng Wang [1,*]

[1] College of Veterinary Medicine, Jilin Provincial Engineering Research Center of Animal Probiotics, Jilin Provincial Key Laboratory of Animal Microecology and Healthy Breeding, Engineering Research Center of Microecological Vaccines (Drugs) for Major Animal Diseases, Ministry of Education, Jilin Agricultural University, Changchun, 130118, China.

* Corresponding authors:

Chun-Feng Wang, College of Animal Science and Technology, College of Veterinary Medicine, Jilin Provincial Engineering Research Center of Animal Probiotics, Jilin Agricultural University, 2888 Xincheng Street, Changchun 130118, China. E-mail: wangchunfeng@jlau.edu.cn, Tel./fax: +86 43184533426.

Wen-Tao Yang, College of Animal Science and Technology, College of Veterinary Medicine, Jilin Provincial Engineering Research Center of Animal Probiotics, Jilin Agricultural University, 2888 Xincheng Street, Changchun 130118, China. E-mail: yangwentao@jlau.edu.cn.

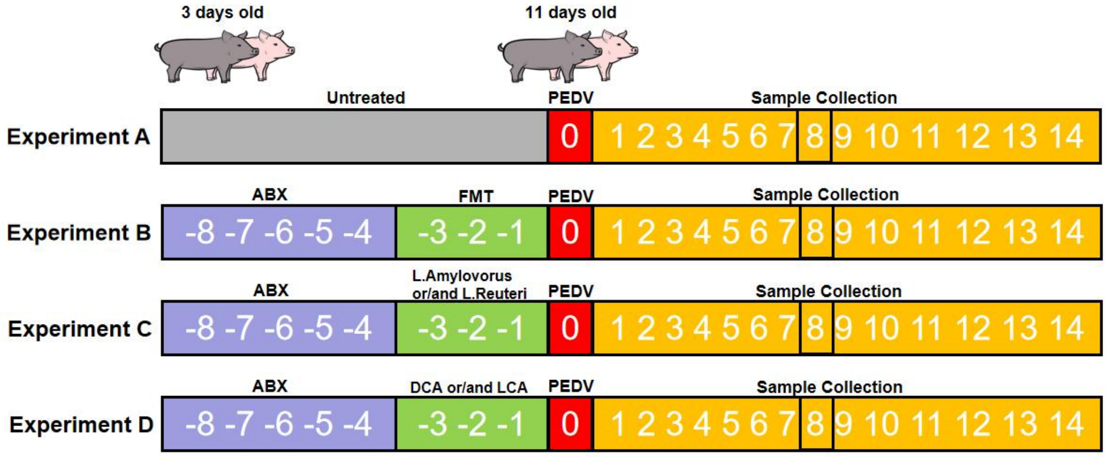

Fig. S1 Time charts for animal experiments A, B, C, and D.

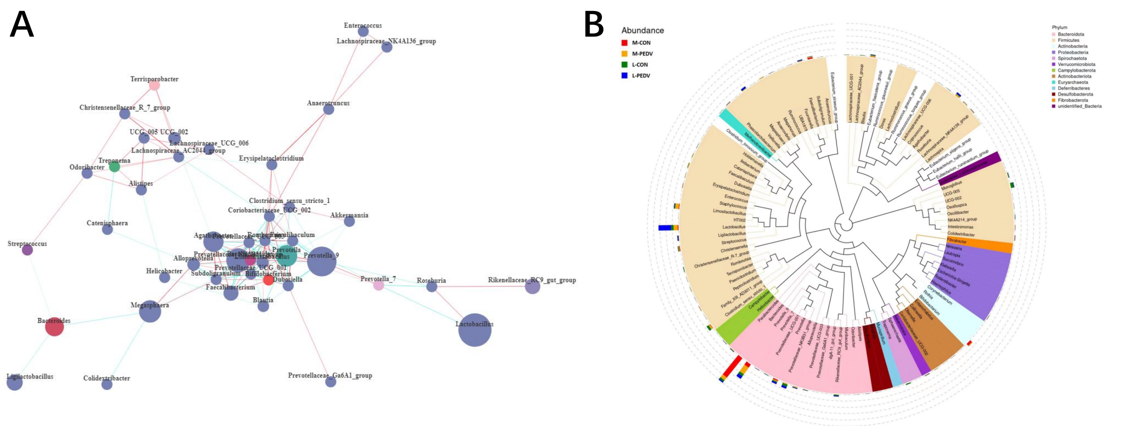

Fig. S2 Genus co-occurrence network and evolutionary tree of intestinal microorganisms in Min pigs and Landrace pigs. (A) The association interactions of the top 50 genera were analysed by calculating correlation indices for all samples. (B) Phylogenetic tree constructed from representative sequences of species at the genus level (top 100).

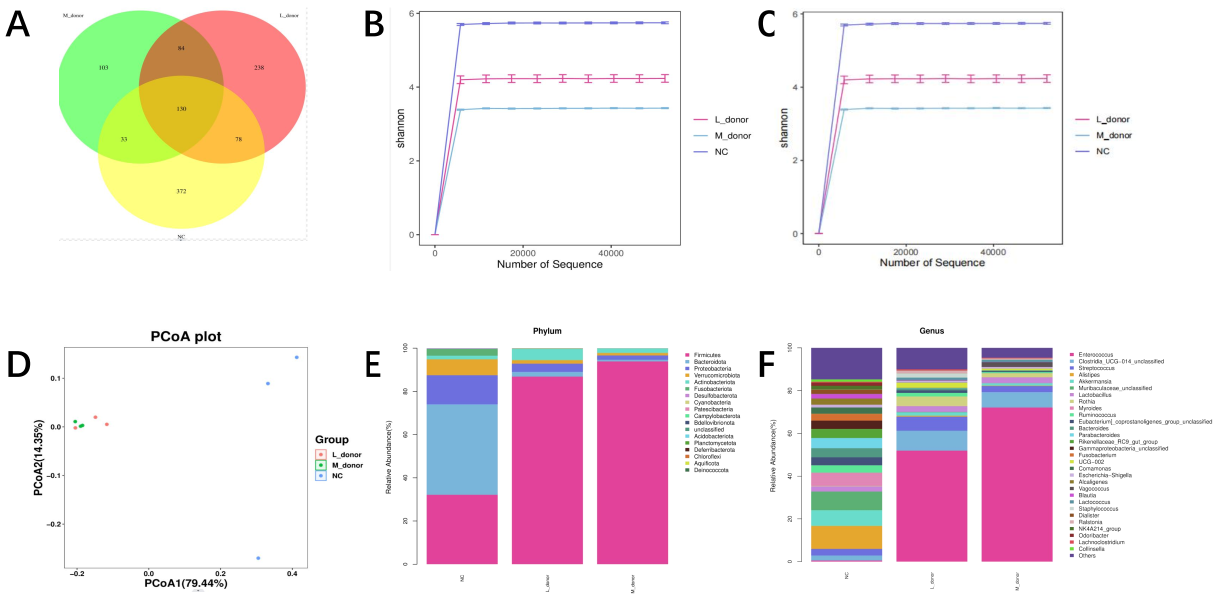

Fig. S3 Validation of the depletion of gut microbes with combination antibiotic treatment. (A) Statistics on the number of OTUs of gut microbes in each group. (B) Chao1 estimate of the total number of species contained in the community samples. (C) Shannon's assessment of the total number of taxa in the sample and their proportions. (D) PCoA based on weighted UniFrac distances to determine changes in microbial structure. The top 10 species at the phylum (E) and genus (F) levels were calculated to assess the species with the highest abundance and their proportions (n=3).

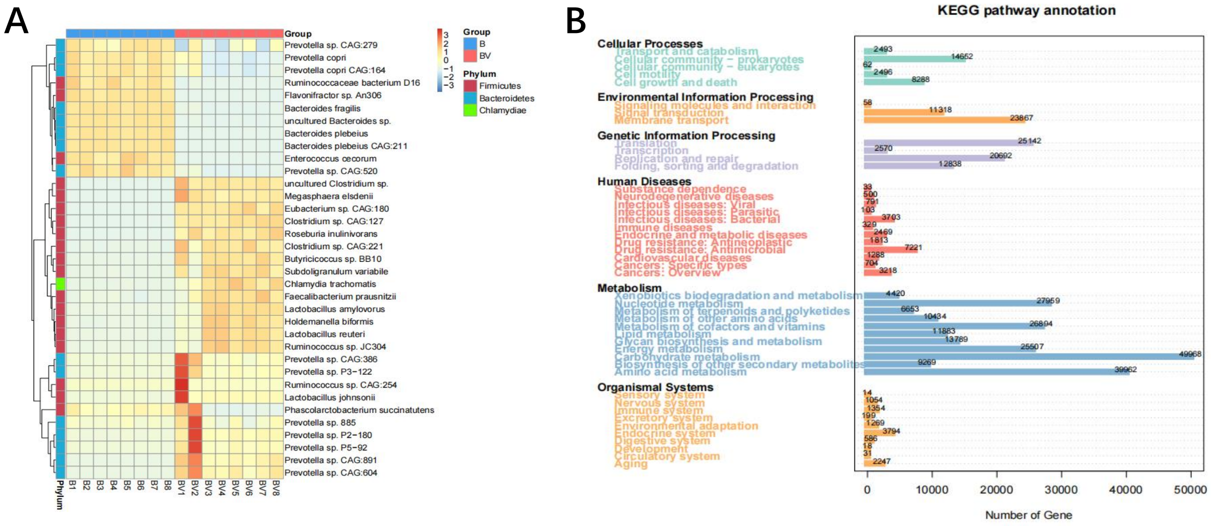

Fig. S4 Macrogenomic analysis of the gut microbes of M-CON and M-PEDV. (A) Clustering analysis of species among the M-CON and M-PEDV gut microbes. (B) Functional enrichment from the KEGG database at six levels.

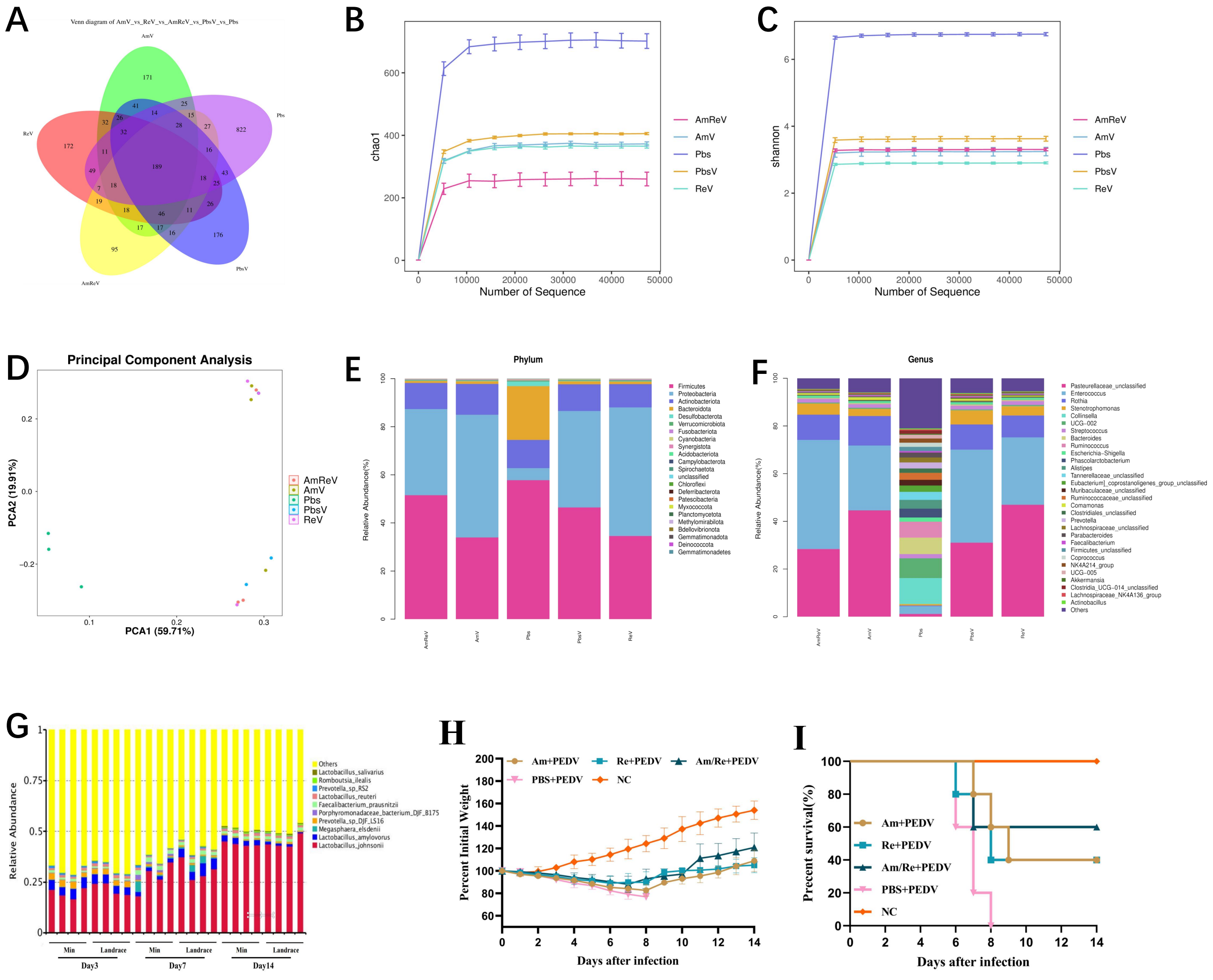

Fig. S5 Intestinal microbial depletion and analysis of bacterial colonization and protective capabilities. (A) Statistics on the number of OTUs of gut microbes in each group. (B) Chao1 estimate of the total number of species contained in the community samples. (C) Shannon's assessment of the total number of taxa in the sample and their proportions. (D) PCoA based on weighted UniFrac distances to determine changes in microbial structure. The top 10 species at the phylum (E) and genus (F) levels were calculated to assess the species with the highest abundance and their proportions (n=3). (G) Analysis of Colonization Potentials of *Lactobacillus reuteri* and *Lactobacillus amylovorus*. (H) Weight Loss Rates in Landrace pigs. (I) Survival Rate Analysis in Landrace pigs.

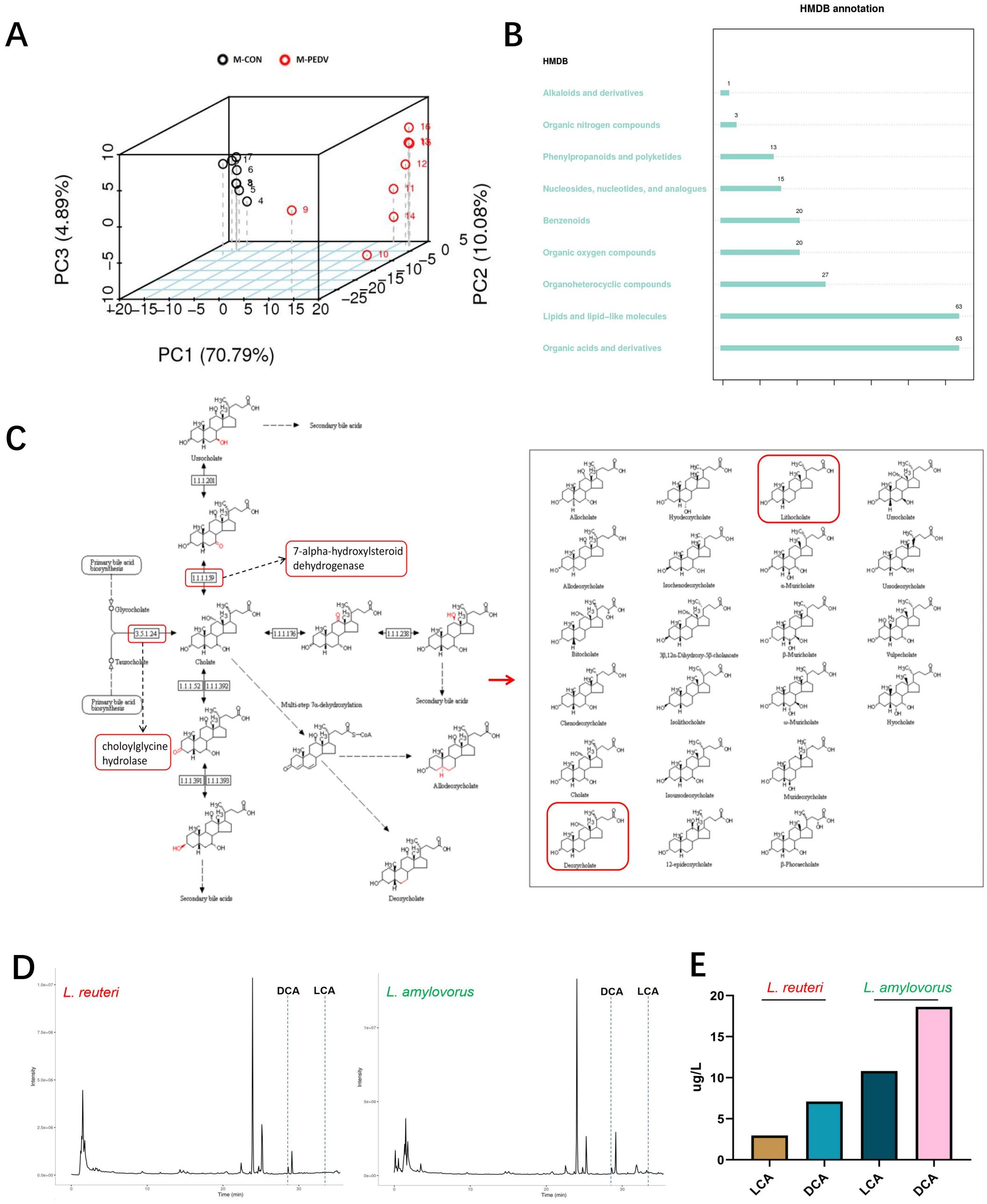

Fig. S6 LC–MS/MS analysis of the M-CON and M-PEDV group faeces. (A) PCA of the intestinal metabolites of the M-CON and M-PEDV groups. (B) Annotation of the Human Metabolome Database (HMDB) of intestinal metabolites. (C) KEGG functional validation of differentially expressed genes in the secondary bile acid synthesis pathway. Qualitative (D) and quantitative (E) Detection of DCA and LCA in the Supernatants of *Lactobacillus reuteri* and *Lactobacillus amylovorus*.

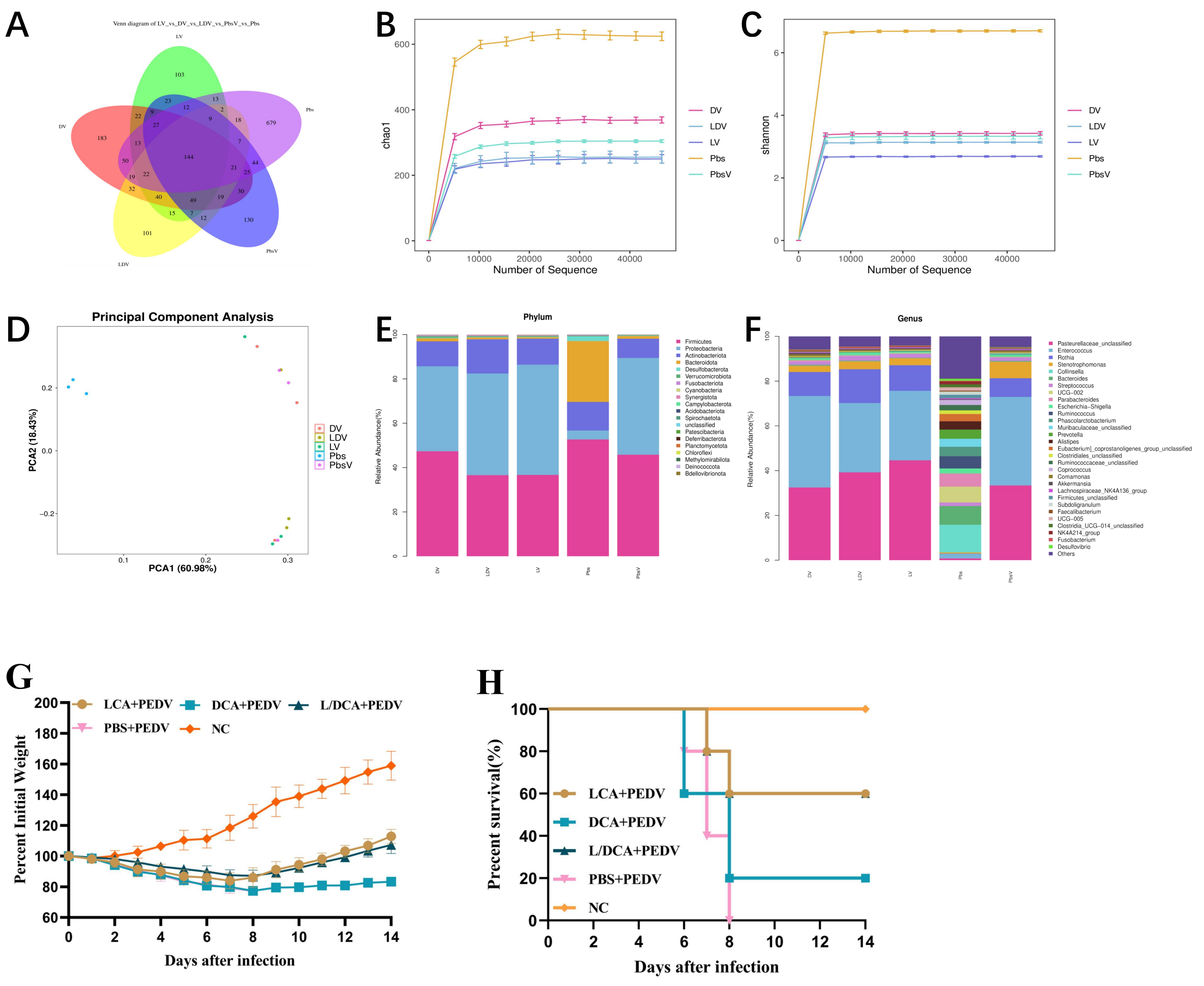

Fig. S7 Analysis of intestinal microbial depletion and strain protective ability. (A) Statistics on the number of OTUs of gut microbes in each group. (B) Chao1 estimates of the total number of species contained in the community samples. (C) Shannon's assessment of the total number of taxa in the sample and their proportions. (D) PCoA based on weighted UniFrac distances to determine changes in microbial structure. The top 10 species at the phylum (E) and genus (F) levels were calculated to assess the species with the highest abundance and their proportions (n=3). (G) Weight Loss Rates in Landrace pigs. (H) Survival Rate Analysis in Landrace pigs.

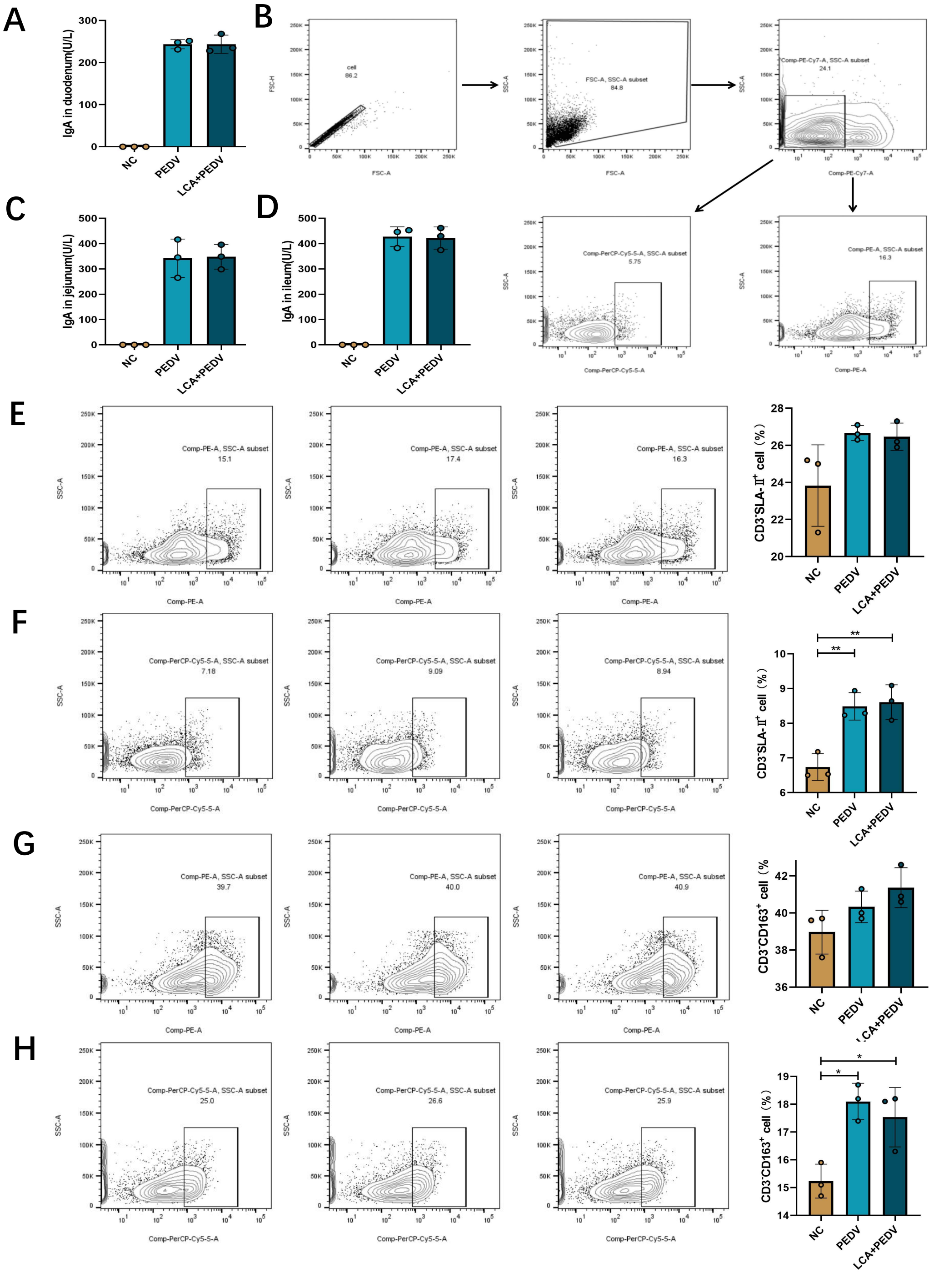

Fig. S8 Effect of LCA on intestinal immune function. IgA expression in the duodenum (A), jejunum (C) and ileum (D) of LCA-treated and untreated piglets after PEDV infection. (n=3/group). (B) Gating strategies for porcine dendritic cells and macrophages. Flow cytometry analysis of dendritic cell (F) and macrophage (H) expression in piglet jejunum and dendritic cell (E) and macrophage (G) expression in porcine mesenteric lymph nodes. (n=3/group). The results are presented as the means ± SDs, and statistical significance was calculated by one-way ANOVA. *$*P < 0.05$; $**P < 0.01$.*

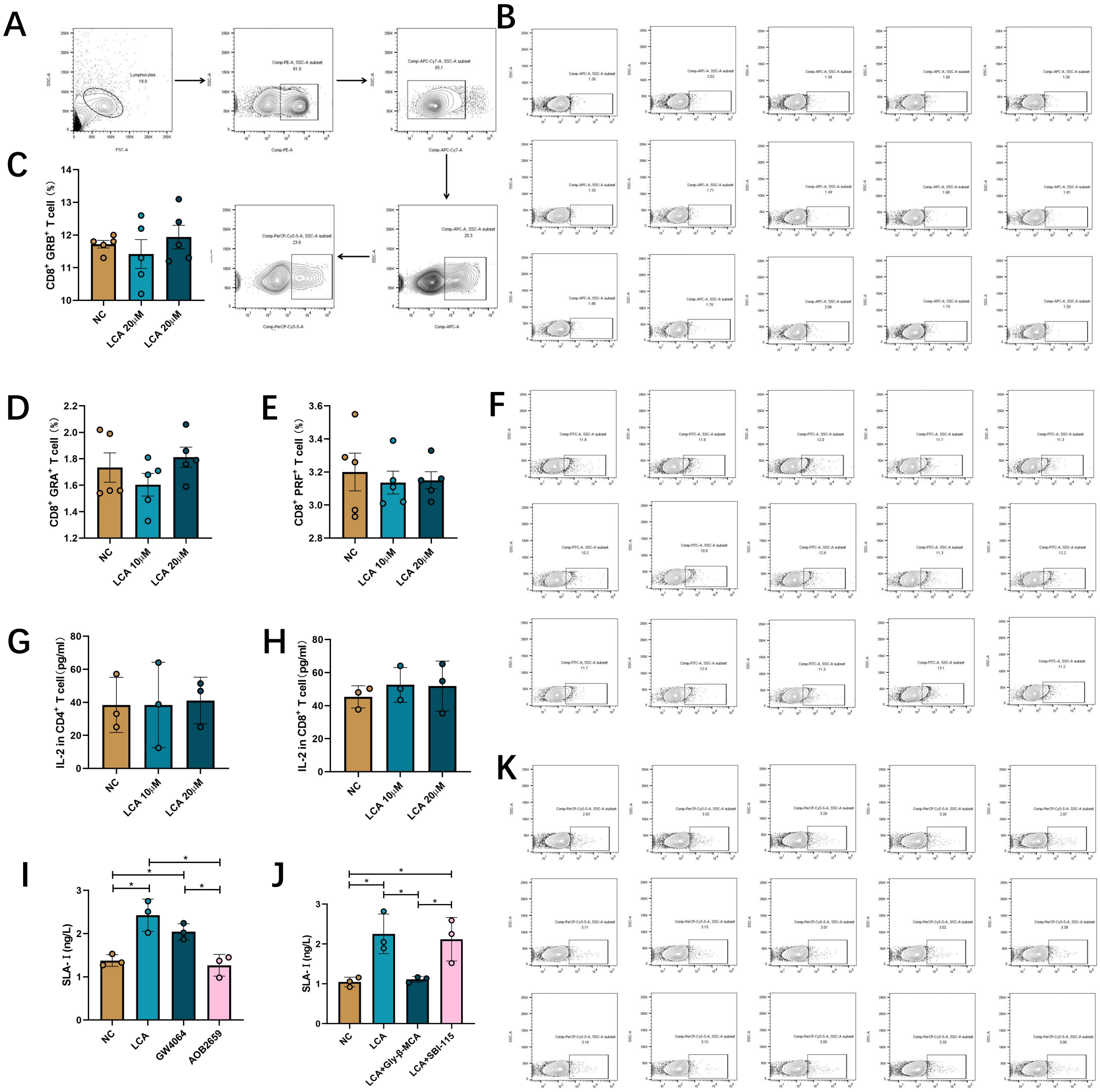

Fig. S9 LCA increases SLA-I expression in porcine intestinal epithelial cells via FXR and thus enhances the killing efficiency of CTLs. (A) Flow cytometry strategy to detect the expression of GZMB, GZMA and PRF in $CD3^{+}CD8^{+}$ T cells. The expression of GZMB (B), GZMA (F) and PRF (K) was detected by flow cytometry after 10/20 μM LCA and anti-CD3/28 treatment of $CD8^{+}$ cells for 24 h, and the percentages of cells with GZMB (C), GZMA (D) and PRF (E) were counted (n=5). Cells treated with 20 μM LCA in concert with anti-CD3/CD28 were isolated from $CD4^{+}$ (G) and $CD8^{+}$ (H) cells by magnetic beads, and the cell supernatants were analysed for IL-2 by ELISA. (J) The expression of SLA-I was detected by ELISA in the presence and absence of SBI-115 and Gly-β-MCA in IPEC-J2 cells treated with 20 μM LCA for 12 h (n=3). (I) SLA-I expression was measured by ELISA in the presence and absence of AOB2659(CCDC) and GW4064 in IPEC-J2 cells treated with 20 μM LCA for 12 h (n=3). The results are presented as the means ± SDs, and statistical significance was calculated by one-way ANOVA. *$P < 0.05$.
